# Interaction-based evolution: how natural selection and nonrandom mutation work together

**DOI:** 10.1186/1745-6150-8-24

**Published:** 2013-10-18

**Authors:** Adi Livnat

**Affiliations:** 1 Department of Biological Sciences, Virginia Tech, Blacksburg, VA, 24061, USA

**Keywords:** Adaptive evolution, Neutral theory, Sex and recombination, Epistasis, Junk DNA, *de novo* genes, Transcriptional promiscuity, Mutation bias, Evolvability

## Abstract

**Background:**

The modern evolutionary synthesis leaves unresolved some of the most fundamental, long-standing questions in evolutionary biology: What is the role of sex in evolution? How does complex adaptation evolve? How can selection operate effectively on genetic interactions? More recently, the molecular biology and genomics revolutions have raised a host of critical new questions, through empirical findings that the modern synthesis fails to explain: for example, the discovery of *de novo* genes; the immense constructive role of transposable elements in evolution; genetic variance and biochemical activity that go far beyond what traditional natural selection can maintain; perplexing cases of molecular parallelism; and more.

**Presentation of the hypothesis:**

Here I address these questions from a unified perspective, by means of a new mechanistic view of evolution that offers a novel connection between selection on the phenotype and genetic evolutionary change (while relying, like the traditional theory, on natural selection as the only source of feedback on the fit between an organism and its environment). I hypothesize that the mutation that is of relevance for the evolution of complex adaptation—while not Lamarckian, or “directed” to increase fitness—is not random, but is instead the outcome of a complex and continually evolving biological process that combines information from multiple loci into one. This allows selection on a fleeting combination of interacting alleles at different loci to have a hereditary effect according to the combination’s fitness.

**Testing and implications of the hypothesis:**

This proposed mechanism addresses the problem of how beneficial genetic interactions can evolve under selection, and also offers an intuitive explanation for the role of sex in evolution, which focuses on sex as the generator of genetic combinations. Importantly, it also implies that genetic variation that has appeared neutral through the lens of traditional theory can actually experience selection on interactions and thus has a much greater adaptive potential than previously considered. Empirical evidence for the proposed mechanism from both molecular evolution and evolution at the organismal level is discussed, and multiple predictions are offered by which it may be tested.

**Reviewers:**

This article was reviewed by Nigel Goldenfeld (nominated by Eugene V. Koonin), Jürgen Brosius and W. Ford Doolittle.

## Background

To explain adaptive evolution, we still use today ideas from the foundations of the modern evolutionary synthesis formed in the 1920s and 1930s. Yet there has been a sea of change in the empirical realities since then. The molecular biology and genomics revolutions have occurred and brought with them fundamental new empirical findings. Some of these findings were simply unexpected from traditional theory and are unengaged by it, including the discovery in the 1960s of far more genetic variance than could be subject to selection according to traditional theory [1,2], and ENCODE’s very recent finding that the majority of the human genome is biochemically active [3]. From the perspective of traditional theory, we are now forced to predict that much of this activity is just “biochemical noise” and not really part of the organism, again because traditional natural selection cannot act on so much evolving matter and for other important reasons [4-8]. Other empirical findings have been more directly challenging. Consider for example *de novo* genes (e.g., [9-13])—genes that presumably have arisen “out of thin air” by a sequence of random mutations that came together into a new functioning gene, including signals for transcription and translation and even alternative splicing [11]. This *de novo* formation takes place even though traditional natural selection could not have acted on this sequence of mutations until the gene was already complete (substantial enough to be active), in clear contradiction with what Jacob justifiably predicted to be impossible [14]. Also challenging to traditional theory are findings of such fundamental significance for our understanding of evolution as the evolutionary organizing of more than 1500 genes into a new genetic network underlying a novel, complex adaptation by transposable elements [15]. Whether for these or other reasons, a sense of curiosity about the new empirical reality has been conveyed by such luminaries as Doolittle [16], Graur and Li [17], Wagner [18], Fedoroff [19], West-Eberhard [20] and others.

In light of these findings, it is commonly assumed that traditional natural selection operates to the extent that it can, and that originally neutral mutations account for anything that selection does not account for. But this modern approach leads to a deep inconsistency. The original idea of natural selection and random mutation, implicit in Fisher’s work [21], was to minimize the amount of “work” done by chance in the evolution of adaptation and let natural selection do the job of evolving an adaptation by pulling out from the noise the supposed slightly beneficial mutations and causing them to accumulate inexorably toward the evolution of adaptation. It is inconsistent to invoke this idea, which attempted to minimize the amount of evolutionary work done by fortuitous chance, while at the same time allowing for an unspecified number of originally neutral mutations to play an inherent role in the evolution of adaptation, as is currently done for example in the case of *de novo* genes. Indeed, there is no quantification of the amount of chance that we call upon to explain the evolution of adaptation (namely the chance that is involved in the arising of accidental mutations and in random genetic drift, to the extent that the latter is invoked)—a deep problem not yet addressed at all by the whole body of population genetics.

This paper holds that the key to solving the fundamental problems brought about by the molecular biology and genomics revolutions is to go back and revisit some fundamental old problems in evolutionary theory that have been open since before even the rise of molecular biology itself. Attending to these old open problems, we may be able to offer a deep change to the core of the theory of natural selection that will reconnect the theory better to the evidence available today. I will begin by discussing two fundamental unresolved problems, namely the role of sex in evolution and how selection on interactions between alleles of different genes can play an evolutionarily constructive role. I will show that, in fact, these two problems are different aspects of one and the same thing.

My general approach will be as follows. I will continue to assume that selection is the only source of feedback on the fit between an organism and its environment. However, I will revisit the question of the nature of the mutation that drives evolution. Here, I will continue to assume that mutation is not Lamarckian, and that a given mutation is not more likely to occur in an environment where it increases fitness than in an environment where it does not [17,22,23]. However, I will show that there is another alternative, which has not been attended to yet, which is neither accidental mutation nor mutation that violates our core assumptions. Revisiting the question of the nature of mutation, I will construct a new theory of how adaptive evolution happens, based on selection, but also on a new connection between selection on the phenotype and genetic evolutionary change. I will show that this approach addresses the unresolved problems of sex and interactions from a unifying perspective, and at the same time begins to propose a mechanism at the point where traditional theory relies only on pure chance. Empirical evidence for and predictions derived from this new mechanism will be discussed for a variety of topics at both the organismal and molecular levels (from plant mating systems and canalization, to molecular parallelism and the nature of mutation, to genetic mechanisms in the sperm cells), with relevance that ultimately goes beyond science to medicine.

The theory will be proposed verbally, and not mathematically, because it is not clear that traditional mathematical tools are immediately suitable for its mathematization. The price of accepting the benefit of unification—where the problems of sex, interactions and the lack of quantification of chance in traditional theory are addressed in one—will be to accept that what we know regarding how evolution happens is merely the tip of the iceberg. An outline of the main points is given in the Summary section.

## Fundamental problems in traditional evolutionary theory: sex and interactions

The most obvious effect of sex is that it creates an exponentially large number of different potential combinations of alleles at different loci—indeed it makes individuals unique. When biologists are asked what the role of sex is in evolution, they often say that from a given number of alleles at different loci it creates this almost endless number of different genetic combinations; and since natural selection operates on genetic variation, this “increased variance” facilitates evolution. But the insufficiency of this explanation is well known to investigators of the evolution of sex and recombination [24]. What is the point of creating, by the shuffling of genes, a variety of genetic combinations that will be tested by natural selection? One may wish to say that, among the many combinations, particularly good ones will be found that would not have existed otherwise. But in saying this, a basic point is forgotten: these combinations of alleles at different loci are not heritable. Just as sex brings them together, so too it breaks them down.

Consistent with this point, the core of the Fisherian theory of adaptive evolution, which forms an essential part of the modern synthesis of evolution [21], is structured in a way that makes these combinations of alleles as complex wholes inessential: following it, population geneticists have often assumed that each allele can have a selective value in and of itself—it can be a “good” or a “bad” mutation (“beneficial” or “deleterious”) with little consideration of the genetic context [25]. This way an allele is “blind” to the particular combinations it goes through. Selection operates statistically on each allele independently of other alleles, because any given allele makes essentially the same additive contribution in different individuals toward the numerical sums that are those individuals’ “fitness values”. Alleles pass each other like ships in the night as they move through the population [26], and the population is treated as a collection of allele frequencies, each for an independent, essentially non-interacting locus [21]. While Fisher did discuss interactions both within and between loci, even in the context of recombination [21], those were not part of his core process of adaptive evolution, which was instead based on independent (or “additive”) effects of separate loci.

However, this way of thinking has left the role of sex a mystery. Notice that the same beneficial or deleterious mutations could have arisen and been favored or disfavored in a sexual as well as an asexual population. By providing a basic mechanism for evolution that works with or without sex, the Fisherian theory has created a view of evolution where sex is not really essential. Since then, investigators only proposed subsidiary and circumscribed benefits that sex may bring on top of an evolutionary mechanism that can work essentially without it (e.g., [26-30]). But all such “bonuses” proposed so far require rather specific conditions [31], and, even considering all of these bonuses together, it is not clear that their collection forms an appealing way of explaining the near-ubiquity of sex [32,33].

Wright never accepted Fisher’s conceptualization of evolution. Wright believed that genes interacted in complex networks and that likewise alleles at different loci must interact with each other to generate any notable evolutionary change [34-36]. The notion of selection acting on each allele in and of itself seemed to him fundamentally insufficient for explaining the evolution of complex adaptation [36]. Note, however, that an interaction between alleles at different loci cannot be persistently selected on, according to the traditional view, precisely because sex disassembles such combinations of alleles, as discussed. Instead of selection, Wright proposed in his shifting balance theory that the basis for an adaptive complex of genes will first arise by chance (after the constituent alleles at different loci have not only arisen by chance, but have also spread by random genetic drift in a given subpopulation), and then natural selection will come to bear on the process by simple (non-interactive) improvements and by helping to spread the constituent alleles from the given subpopulation to other subpopulations through migrants [34,35]. This theory required stringent conditions on the population structure [37,38], attempted to obtain the basis for a new complex adaptation by pure chance, and has not been uniformly accepted [38,39]. Thus, in distinction from selection on separate genetic effects and the supposed chance formation of the basis for beneficial genetic interactions by random genetic drift, we still do not have a theory for how selection on genetic interactions can be at the core of the adaptive evolutionary process.

There are multiple ways to derive the theory presented here, but the one described below begins with the problem of sex and interactions just mentioned. In accordance with the long-standing intuition of biologists, I will argue that the essential thing about sex is that it generates combinations of alleles at different loci; indeed I will argue more: that these combinations are a matter of necessity for evolution. From the traditional theory, this cannot be, because these transient combinations cannot be inherited. But we will soon realize that they can, though not in the traditional way. This will take a sweeping change of outlook, which at first appears to be itself impossible: I will posit that mutation is nonrandom, and show that this solves the problem from the traditional theory that combinations of alleles cannot have a lasting effect. This appears impossible at first because we are correctly trained to avoid Lamarckian transmission [40] and Lamarckian “directed mutation” as possible explanations for evolution at the general level [22,23]. But the nonrandom mutation discussed here will not be of these kinds. The “nonrandomness” I will refer to is emphatically not the one where mutation is more likely to occur in an environment where it increases fitness, and is therefore not the one disallowed by traditional theory [17,22,23].

## Selection on interactions can drive evolution when mutation is nonrandom

Let us develop the concept of nonrandom mutation here carefully from square one. By “nonrandom mutation” we will mean that the mutation that drives evolution is not accidental—it is not an unintended disruption of the genetic code, caused for example by external agents or by oxidative stress (although mutations of such kinds do happen and can lead to disease). We will take this to mean that the mutation that drives evolution is the result of an organic process that belongs to the organism.

If so, then like all other biological processes that belong to the organism, this process must be specified by the genes. These genes interact, as genes always interact in the determination of a trait, except that, while a classical trait is something that serves in the survival and reproduction of the organism, here we are talking about a trait whose end result is genetic change. While genes interact and lead to a classical trait like the ear, here genes interact and cause genetic change.

**Figure 1:**
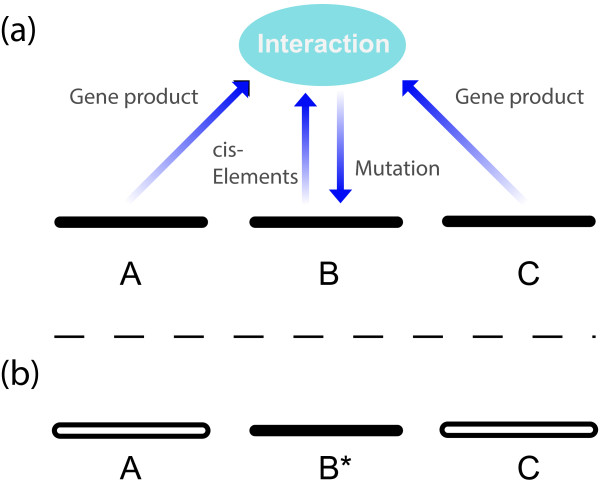
**Mutation as a biological process. a)** Mutation as a biological process means that genes interact in the determination of mutation. In the schematic figure, information from three different loci (A, B and C) comes together, through cis-acting elements and trans-acting factors, to affect the probability and nature of a genetic change in one of these loci (B). Inputs into this mutational process are shown by the annotated arrows. The downward arrow represents the writing of mutation, for example by components of the so-called “error-repair” machinery, here not restoring but changing the genetic state from what it was previously. In reality, many more pieces of information than depicted here for simplicity may be involved. **b)** After meiosis, the changed locus (B*) carries in it an information-signature from the combination that participated in the generation of the change, and thus allows the combination as a whole to have a lasting effect, even though its components are no longer all present.

Given that genes interact in the determination of genetic change, and keeping the assumption that their alleles interact, this means that the mutation that drives evolution is a process that combines information from alleles at multiple loci and writes the result of the combination operation into one locus—the locus being changed by mutation (Figure 1a). (Also if multiple loci are changed at once, information is combined from multiple loci to enact these multiple changes.) By combining information from alleles at multiple loci into one locus, this operation creates from the combination of alleles a piece of information that is not broken by the sexual shuffling of the genes, and is therefore heritable (Figure 1b). (It creates an allele, and this is an elementary unit for the shuffling; the shuffling breaks only combinations of alleles). This means that combinations of alleles at different loci do have an effect that lasts through the shuffling: they transmit information to future generations through the mutations that are derived from them.

This general-level point is as simple as it is crucial: if mutation is nonrandom, then selection on interactions has a hereditary effect. While selection on combinations means that successful combinations survive and reproduce preferentially, the writing of mutation takes these successful combinations and makes heritable mutations from them that will be transmitted to the next generation. Thus, natural selection on genetic combinations and nonrandom mutation work together.

Interestingly, there have always been only two main ways of thinking about adaptive evolution (though more if we consider smaller variants and less influential streams): One has been the Darwinian theory of natural selection, which was turned into the neo-Darwinian theory of natural selection and random mutation (ns/rm) in the 1920s and 1930s. In this theory, differential survival and reproduction is the source of feedback that allows the fit between an organism and its environment. The other has been the Lamarckian-transmissionist one, which holds that the organism is somehow able to sense what is needed for improvement in terms of the fit to the environment and then is able to change the hereditary material in a way that improves this fit, thus transmitting the improvement to the next generation. This Lamarckian-transmissionist option is not only impossible as a general-level explanation for evolution [40], but, interestingly, if it were possible, its action would have rendered selection redundant [41]. Therefore, the Lamarckian kind of nonrandom mutation on the one hand, and natural selection on the other hand, are rival hypotheses. We can now see that the theory presented here is a third alternative, distinct from the above two. The nonrandom mutation considered here and natural selection are complementary, in diametric opposition to the above rivalry. Differential survival and reproduction is the source of feedback on organismal fit to the environment. Nonrandom mutation collects this feedback in a manner that allows natural selection to act on genetic interactions. Thus, selection on the organism as a unified whole is possible.

The theory just proposed connects empirical facts at a deep level. It explains sex while making a substantial statement about the empirical nature of mutation: the mutation that drives evolution is nonrandom^**a**^—it is an organic process that belongs to the organism. Evidence and predictions regarding this statement will be discussed later (see the section “Evidence from and predictions for molecular evolution”), after further theory is developed that will make them clearer.

In the following sections I will discuss the prevalence, origin and maintenance of sex, the nature of the evolution of complex adaptation at the phenotypic level, and how they connect to the above. The reader who is primarily interested in the molecular side of this theory may skip to the section “A more detailed look into the new theory”.

### Sex as a matter of necessity for evolution

Having described the core of the theory we can now expand on our empirical view. I use Barton and Charlesworth’s [24] evolutionary definition of sex as the shuffling of genes among individuals that leads to the creation of offspring that are genetically different from their parents. According to this most basic evolutionary-biological definition, sex is nearly universal [24]: it occurs in plants and animals by syngamy, in fungi via the fusion of hyphae and in bacteria by conjugation and other means [33,42]. Many species are capable of reproducing both sexually and asexually, but because their bouts of sexual reproduction keep their genes shuffled, they will be considered sexual here. We will consider “asexual” those species in which the shuffling of genes does not occur. Those are the obligate asexuals.

Several important facts can now be pointed out. First, obligate asexuals are very rare. For example, Vrijenhoek [43] estimated that about 1 in 1000 animal species is an obligate asexual. Second, they appear to be headed toward ultimate extinction without leaving descendant species behind. This point has been inferred from their phylogenetic distribution: they inhabit small, recent, sparsely distributed twigs on the tree of life, which is consistent with the idea that they occasionally arise as terminal offshoots from sexual species (sexuals are the source and asexuals are the sink) [44-48]. Indeed, their structure shows that they are recent derivations from sexual ancestors: selfing plants still have reproductive structures that have served them in sexual reproduction in recent evolutionary times [49]. Given this evidence (see further discussion in the next section), we can infer that the immortal part of the tree of life is sexual.

Interestingly, and consistent with the above, Stebbins concluded from extensive studies of plant morphology that asexuals are incapable of true evolutionary innovation [49]. In accord with Stebbins [49], they have often been called “evolutionary dead ends”. We must also ponder the great extent of adaptive structure and effort devoted to implementing the shuffling of genes throughout the biological world. From flowers to butterflies to human behavior, we do not need science to tell us that sex forms an important part of the biological world. Indeed, it is intertwined with biological structure and function down to the molecular level, where meiosis involves extremely complex molecular machinery that implements the shuffling of genes.

With these facts in mind, we can now obtain a high-level insight on sex by comparing it to its “peer” biological phenomena. What other phenomena are ubiquitous across the immortal part of the tree of life? Sex can barely be matched in terms of this ubiquity and importance. In this part of the tree of life, it can only be matched by such things as reproduction per se, metabolism in general, and the existence of the genetic code itself. Importantly, these phenomena are parts of the fundamental framework of life. They are not there because their “benefits outweigh their costs”; they are simply necessary. They are part of the definition of the process, as we do not contemplate biological evolution without some kind of a conveyor of hereditary information, without reproduction or metabolism. In accordance with the evidence, these are the “peer phenomena” of sex; and in keeping with a parsimonious picture, I hold that like its peer phenomena, sex is also a matter of necessity for evolution, a part of the infrastructural group.

Now note that the principle that sex is a matter of necessity for evolution, based on empirical facts, is consistent with the new theory of evolution just proposed, but is inconsistent with traditional theory. It is consistent with the new theory because this theory argues that genetic combinations are a matter of necessity for evolution (selection operates on them), and sex creates these combinations. It is inconsistent with traditional theory as already discussed—the Fisherian theory offers a way of understanding evolution that takes sex conceptually out of the level of the essentials.

### A prediction following work from Meselson’s lab

The contrast just mentioned renders particularly important the empirical question surrounding the putative ancient asexuals. It has been thought for a while that some asexuals may have evolved and diversified substantially, giving rise to asexual clades, the most famous example being the bdelloid rotifers. Since no one has observed these minute organisms in the act, they have been thought to have evolved and diversified asexually for more than 35 million years, giving rise to 4 orders, 18 genera and 363 “species” according to one report [50]. The possibility that there are such exceptions to the rule of asexuals as dead-ends has not been a fundamental problem for the traditional theory. Under the traditional theory, sex is not part of the evolutionary infrastructure but a bonus for which various separate reasons have been proposed, each with its own specific conditions required. Thus, if an ancient and diversified asexual clade is observed, it can always be argued that it does not satisfy any of the requirements for sex without violating the core of traditional theory (see Judson and Normark [50] for a discussion of this topic). Indeed, the problem lies more in the other direction: one may ask why there are not many more putative ancient asexuals, as no clarity is given from traditional theory over why the specific conditions required for the various bonuses proposed would sum up to cover nearly all of nature.

However, for the theory presented here, the existence of an ancient, diversified asexual clade would be a fatal problem; because it would show that true evolution can happen without sex, thus refuting the new theory. This raises a prediction. According to the theory presented here, all the putative diversified asexual clades are false examples in the following sense: if their members have undergone substantial adaptive evolution and diversification, they have done so in a sexual state. Two possibilities that are in accord with this prediction are that most of their members are still sexual today, or that a sexual core exists [51] from which asexuals are continually spun off due to hybridization or other reasons. According to both of these possibilities, even if we have not yet observed mechanisms of sexual shuffling of genes in these organisms, they are out there to be found, and so if we look for them we will find them, according to this theory.

It is of interest, therefore, that Meselson recently reported [52,53] that, having set to prove once and for all that the bdelloid rotifers are asexual, his lab seemed to have found the opposite: genetic analysis shows homologous gene shuffling in bdelloid rotifers. However, we still do not know how they do it—by what mechanisms they exchange genes or what triggers their elusive bouts of gene exchange. Assuming this result, not yet published at the time of writing, holds, one prediction of the theory presented here is already underway to being confirmed. Beyond this case, there are a couple of dozen other cases of putative ancient asexual clades [50], which provide opportunity to test, and refute, this theory.

### Sex predates asex

As soon as one proposes the principle that sex is a matter of necessity for evolution, a question comes up: If evolution started in an asexual state, with sex emerging at a later point, then evolution was already taking place before sex. This in turn would mean that sex is not necessary for evolution.

Indeed, discussions of the origin of sex have often been couched implicitly or explicitly in terms of “why did sex arise?” (presumably from asex) and “what benefit did it bring that gave it the advantage and led to its prevalence?” [24,46]. This discourse shows that there has been a tacit assumption that sex arose from asex, and that it outcompeted asex because its “advantages” outweighed its “costs”. If it arose from asex, this implies that it is not a matter of necessity, as just mentioned; and if it succeeded because its “benefits outweighed its costs”, then it is not a matter of necessity—it is not a member of the infrastructural group, to which this balance of costs and benefits is not applied.

But why have we been making this tacit assumption? One reason might be that sex appears to be more complex than asex, so it seems as though asex should have come first, and sex should have been derived from it. But the fact that the sexual mechanisms of today appear complex does not mean that they have always been so. In fact, if we ask ourselves what sex is at the most basic level, we will find that it is merely the mixing of genetic material. So even if we push the conversation all the way back to the so-called “primordial soup”—an era of utter speculation—we will find no reason to insist that this primordial soup must have been asexual. The free mixing of compounds that the image of the “primordial soup” entails could just as well have been a “sexual beginning”. Indeed, all that we see from present evidence is that asexuals arise from sexuals; and that the asexuals are less complex than the sexuals because they are “broken sexuals”—sexuals with a missing piece in them. The hidden assumption that life started asexually must be exposed, because it has no empirical basis. Instead, the theory presented here is supportive of pioneering theories of Woese [54], Brosius [55,56] and Vetsigian et al. [57] on rampant gene exchange in early life, and of Williams’s and others’ views that sex is original [48].

### An adaptation evolves by convergence on the population level

I will now describe the second point of the theory: if evolution is based on interactions, then a trait arises not by sequential addition of one change at a time, each serviceable on its own, but by gradual stabilization of the trait as a complex whole—by a process of convergence on the population level, as defined below.

For a trait to be part of the long-term process of adaptive evolution—in such a manner that it is not transient, but rather further adaptive evolution can be based on it—we expect it to ultimately belong to all the individuals in the population or species of interest (even if we are interested only in the population of individuals of a particular morph or sex). How does a new trait come to be shared by all individuals in a given population? The Fisherian theory has a ready mechanism for it: A new allele arises by random mutation that has a phenotypic meaning and a fitness value in and of itself. It makes the same change in the phenotype regardless of the particular individual genetic combination it is in. If this allele is “beneficial”, it will spread by traditional natural selection from the one individual in which it arose to the many, bringing along with it the change that it causes in the phenotype to the whole population. Thus the population comes to share this change. Then, another beneficial allele will arise in some individual, spreading and bringing its own change to all, and so on and so forth. It is very easy to see here how the population comes to share a new trait.

However, if evolution is based on interactions, and interactions are not heritable in the same way that a Fisherian allele is, how does the population come to share a new trait?

Let us define a trait on a population level as something that belongs to all individuals in the population or species of interest and thus does not change much as we move from one generation to the next through the sexual shuffling. (Note that this definition defines the trait on a population-level. One can still talk about a “trait” that belongs to an individual, or an individual variant. But an evolved adaptation is shared among individuals, and is captured by our definition of a trait). Now, consider the genetic differences between individuals in a population at some arbitrary generation, generation *t*_0_. Over the generations, some of the *t*_0_ alleles become fixed, others become extinct, and thus the genetic differences of *t*_0_ gradually disappear (even as they give rise to new differences in the meantime in accord with the theory presented here, as will be seen in the next section). This means that the effect of the sexual shuffling on the phenotype that is due to the interactions between these genetic differences of *t*_0_ gradually becomes smaller. This means that the parents of generation *t*_*x*_ and their offspring in generation *t*_*x*+1_ gradually become more similar to each other as far as the phenotypic differences caused by the genetic variance of *t*_0_ are concerned, as *x* is increased. The differences of *t*_0_ have been removed, and something has become stable in the genes.

We must conclude from the above that the evolution of an adaptation occurs by convergence on the level of the population as a whole. It is a process of stabilization. (I use the word “convergence” here not in its evolutionary jargon meaning but in its dictionary meaning of “moving toward union or uniformity” [58] or, in the verb form “converge”, “gradually change so as to become similar or develop something in common”, or “come together from different directions so as eventually to meet” [59]). Interestingly, this gradual stabilization on the population level in the long term of what previously used to vary fits much better with Darwin’s own observations on variance [60] than the so-called “neo-Darwinian” (the traditional) theory does.

Thus, as to the question of how a new trait comes to be shared, we see that, in the present theory, alleles still spread in the population. At the end of a time period, many alleles would have reached from the individuals in which they arose to the entire population. These alleles represent a certain amount of information that has come to be shared by all, and thus a new trait can be shared. The difference from Fisher’s additive-effect–based theory is that each allele does not have its own phenotypic meaning and the trait does not arise in a one-at-a-time fashion by the additive accumulation of independent steps. Instead, the meaning comes from the whole of those interacting genetic changes taken together.

### The writing of mutation provides the physical basis of convergence

Note that we have just derived the fact that evolution happens by convergence on the level of the population as a whole from the fact that evolution is based on interactions (that combinations of alleles, and sex, are matters of necessity for evolution). Interestingly, though the writing of mutations was derived independently from the same fact, we can now see that it helps us understand the physical basis of convergence. Thus, the two independent derivations come together.

**Figure 2:**
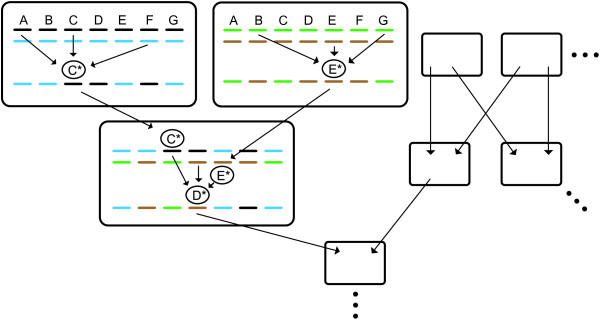
**A population-level view.** If mutational writing is a biological process, then information flows over the generations from many ancestral combinations into each descendant, and from many loci into each of many single loci, forming a network of information flow across the genome over time. Mutational writing events are shown for the sake of demonstration in three individuals (two parents and an offspring, large boxes), but occur also in other genes and other individuals (to avoid clutter, only one writing event per individual is shown).

I argued that the writing of mutations combines multiple pieces of information from alleles at multiple loci as it puts them into one mutation—into one locus. Now, many such writing acts take place across the genome and over the generations, and a new allele that is the outcome of the writing in one generation is part of the input into the writing in another (it is at the tip of the writing “funnel” in one generation and part of the funnel’s base in another). Thus, if we take the many writing acts across the genome and over the generations together, we can see that each allele in a late generation traces its origin to many alleles at different loci in a sufficiently remote early generation (much like an individual in a sexual population traces its origin to many ancestors in a sufficiently remote early generation) (Figure 2). This means that, the farther we get in time from the early generation, the more the basis of information in the early generation comes to be shared by individuals. In other words, the population is converging, and the writing, by actually putting information from different individual combinations (and from different loci) together, provides the basis for this convergence.

Note that the writing acts are connected in a network: they represent a flow of information over the generations from many loci into one and from one to the many. This flow converts information from a state where it is unstable under the shuffling of genes to a state where it is stable under this shuffling, and the result is the writing of a genetic network.

### Obligate asexuality evolves by “breakage”

The empirical evidence fitting with the principle that sex is a matter of necessity for evolution provides empirical support for my theory, as discussed. Additional evidence from the topic of sex comes from the question of its maintenance. The reduction principle [32,61-63]—one of the most robust findings of theoretical population genetics in the 20^th^ century—shows that, in a world consistent with the modern synthetic view, it would be hard for the sexual recombination rate to be maintained rather than be reduced. This has been an important, negative result showing a difficulty in explaining sex in a straightforward manner from a traditional perspective.

However, if sex is necessary for the evolution of complex adaptation, and this evolution happens by convergence, then there is a barrier to evolving obligate asexuality, because the closer the population gets to obligate asexuality, the less it is able to further evolve adaptively in this direction (or in any direction). This leads to the interesting prediction that the process of adaptive evolution toward asexuality will slowly grind to a halt and will not reach the pure asexual state. Therefore, if there are obligate asexual species, it is not long-term adaptive evolution that led to them, but some kind of “breakage” of the sexual mechanism.

This constitutes a very different approach than available so far to the question of how sex is “maintained” despite its “costs” [24]. I have claimed earlier that it is incorrect to discuss sex as something whose “costs” and “benefits” determine its existence, because it is a matter of necessity. And here I claim that it is not actively “maintained”, but rather no substantial adaptive evolution occurs without it, and so obligate asexuality cannot gradually and adaptively evolve. It can only arise non-adaptively. The rest of this section will consider evidence and predictions regarding this point.

Note that, among vertebrates, all known unisexual lineages according to Avise [64] have arisen from hybridizations, which is a sudden, breakage event. Indeed, it is thought that hybridization probably disrupted the meiotic operations by reducing chromosomal homology enough to disrupt synapsis [64-67]. While this fact is in accord with the new theory as just stated, in retrospect, one may try to argue that it is consistent with the traditional one too, because if sex is already well established in two separate sexes, then it is hard to see how it will evolve into asex except by breakage. Let us therefore take the battle to the flowering plants: there, most species are capable of both selfing and outcrossing (selfing being akin to asexuality). According to traditional theory, the entire range from pure selfing to pure outcrossing is open to them, and adaptive evolution should be able to push species all the way to pure selfing or pure outcrossing [68]. Indeed, from that theory, based on inbreeding considerations, the paper that initiated the modern interest in this field predicted that pure selfing and pure outcrossing are the only stable equilibria under adaptive evolution [68]. But that approach gives no clear reason why there are overwhelmingly more species at the outcrossing end of the spectrum than at the selfing end [69-74]. This empirical fact supports the theory proposed here while standing uncomfortably with the traditional one. That is, according to my theory, evolution in a mixed selfing-outcrossing system is possible, but pure selfing can only be reached by breakage. Pure selfing is rare because it requires breakage, which can occur only under very specific conditions. (In the unisexual vertebrates, for example, it has been argued that the hybridizing species need to be genetically close enough to produce a viable hybrid but far enough to disrupt meiosis [43,75] and/or satisfy more specific restrictions [76].)

To be sure, other explanations have been offered in the plant-mating literature for the lack of pure asexuals (e.g., [77-84]), but the explanation proposed here is both simpler and more general. Indeed, it predicts residual outcrossing in regular biparental inbreeding animal species, which goes beyond hermaphrodites.

Because the new theory holds that obligate asexuality is arrived at by breakage, it predicts the lack of fine-tuned adaptations ensuring obligate asexuality. In contrast, from traditional theory, one would expect adaptations for pure asex in like manner as for pure sex. This suddenly renders of particular importance the empirical question of what obligate selfers are like. Pannell [85] mentions two notable examples of obligate selfers. One example involves the loss of males in populations or species of androdioecious animals. In these animals, such as the mangrove killifish (*Kryptolebias marmoratus*) or *Caenorhabditis elegans *[85], individuals are either male or hermaphrodites that can mate with males but not with each other [86-88]. Loss of males in such a situation leads to obligate asexuality. But notice that this loss of males is not a long-term adaptive evolutionary process, but a situational event. It can occur, for example, due to the absence of males from a founding population. Even if it is assumed, hypothetically, that the loss of males is due to selection favoring hermaphrodites and leading to the loss of an allele for male determination [89], this is still a short-term, population dynamical process where no evolution of new adaptations or structures occurs. Only the simple loss of preexisting parts of the sexual machinery occurs, which does not contradict the theory proposed here. This case can be classified as a breakage event, broadly construed, and does not provide an example of gradual adaptive evolution of new structures.

The other example concerns the cleistogamous plants, and provides a test-case for the theory proposed here. In these plants, some flowers never open, and only selfing can occur within them. Most cleistogamous species have both closed and open flowers [90-92], and it has been suggested that the closed ones provide a cheap supply of seeds and reproductive assurance under unfavorable conditions (see [90] and references therein). The closed flowers have adaptive modifications to facilitate selfing [90,91] and, according to the present theory, these adaptive modifications can evolve in a mixed mating state, i.e., while the species has both open and closed flowers and reproduction both by selfing and by outcrossing occurs. Of interest are the ∼10% of cleistogamous species that have only closed flowers at present [92]. If their complete lack of open flowers arose by adaptive evolution, it would refute the present theory; if it arose by breakage it would support it.

It is conceivably possible to try to distinguish empirically whether complete cleistogamous species evolved by adaptive evolution or by breakage. We know that the flowers of cleistogamous plants are generally sensitive to the environmental conditions, such that they often remain closed under unfavorable environments and open under favorable environments [90-92]. Thus, it is possible that the loss of an environmental condition causes a transition from partial to complete cleistogamy without adaptive evolution occurring. Furthermore, genetic deterioration, perhaps even due to insufficient pollination in a mixed-mating state, could be another reason for the failure of flowers to open. It is interesting that the failure of flowers to open could be the reason for the switch to complete cleistogamy, because it means that the biological nature of this prominent case of obligate asexuality makes an allowance for the present theory, whereas from traditional theory there is no reason why the nature of the situation would be as it is. (The point is the mechanistic nature of an adaptation and the breaking of it: the breaking of the process of the opening of a flower in a partially cleistogamous species will make the flower not open—it will make cleistogamy complete. Compare the situation to that of the true, complex and fine-tuned adaptation that is self-incompatibility [93,94]. There, breakage would only lead to less, not more, of what that adaptation provides.)

Detailed studies of the history and nature of adaptation of complete cleistogamous species and other pure asexuals can serve as empirical test cases of the theory presented here. At most, this theory may be consistent with very limited evolutionary modifications in a pure asexual state, perhaps due to residual writing activity inherited from sexual ancestors, and those limited modifications may tend to show simplification and destruction of parts. But it does not allow for the evolution of a novel, complex adaptation in a purely asexual state. In contrast, observations of breakage of various kinds in the evolution of pure asexuality would support the theory presented here. Note that the fact that the examples considered either explicitly fit or potentially fit with breakage is consistent with the present theory and has no general explanation from the traditional theory.

To clarify, my theory does not argue that no degree of selfing can evolve adaptively [95,96]. It only argues that pure asex cannot evolve in this manner. One can think of it as follows: according to my theory, if there is an “objective” that an evolving adaptation maximizes, it is the extent of participation in the sexual population; not simply the expected number of surviving offspring without regard to their sexuality. Under the right conditions, a high rate of selfing may maximize the bottom-line participation of a lineage in the sexual population; but pure selfing fails in this objective. The conditions that have been found to be empirically associated with increased selfing may be interpreted from this perspective without further change. The situation is analogous to the choice between saving versus spending in economic models [97], or the choice between investing in survival vs. reproduction in life history theory [98]. Given the right conditions, saving more can lead to an overall greater consumption over time, and investing more in survival may lead to a greater number of surviving offspring at the bottom line. But it is not a solution to spend nothing; it is not a solution to not reproduce at all; and by analogy, pure asex is not an outcome of adaptive evolution, according to the theory presented here.

In sum, it can be concluded that the entire traditional conceptualization of sex needs to be changed: 

a) Sex is necessary for evolution, it is not a “bonus”.

b) Sex cannot evolve from asex and never did.

c) Sex (as opposed to pure asex) is not actively maintained under some cost–benefit balance as previously discussed. Rather, pure asex arises only through breakage and never through gradual adaptive evolution.

### Empirically, the process of the evolution of adaptation looks like convergence

The process of convergence described here fits better than traditional theory with what the evolution of adaptation looks like empirically. A single example will be given here, from one of the best studied cases of the phenotypic evolution of complex adaptation [99].

Sand wasps (Bembicinae; previously Nyssoninae) dig a long, narrow tunnel into the ground at the end of which they construct a cell or a complex of cells where they lay their eggs and provision their larvae. Their parasites, certain groups of flies and parasitoid wasps, aggressively seek their nests to lay their own eggs in them, for example by flying over the ground, constantly tapping the soil with their antennae. In many sand wasp species, a behavior has evolved where the sand wasp digs one or more false burrows that extend from nearby the real nest entrance into the ground. They leave these decoys’ entrances open, and the real entrance closed^**b**^.

Comparative ethological studies [99-101] show a range of species from primitive to advanced in this behavior of constructing false burrows. In species more primitive in this behavior, the false burrows are short and unstable, and can be easily destroyed by the elements. In species more advanced in this behavior, the false burrows are long and pronounced and, in some of these species, they are actively maintained (that is, restored if disturbed). Importantly, in the species that are more primitive in this behavior, the construction of the decoy burrows is highly variable among individuals—it is disorganized: it varies in terms of whether or not a false burrow appears, how pronounced it is, where it appears spatially, when it appears in the course of nest construction, and the digging that causes it can be scattered over time—it is unfocused. In brief, the whole operation is crude, or “fuzzy” (but it is there as a whole). Whereas, in the advanced species, individual variation in the behavior is far reduced, and the overall pattern of construction is much more stable. The tunnels appear regularly and are pronounced, and they generally have a time and a place of focused construction. In a word, the operation is sharp, like clockwork; and it is far more similar among individuals. Since it is standard to infer from a transitional series of contemporary variants to the evolutionary process of one variant^**c**^, this evidence suggests that the process of the evolution of this complex adaptation has been a process of convergence on the population level—a process of stabilization—where the trait as a whole evolved from a state of high variance to a state of low variance.

This stabilization and sharpening of the trait as a whole clearly fits with the process of convergence predicted by the theory presented here, but it is not inherent to traditional theory. Investigators have tried to explain stabilization without invoking nontraditional theory by invoking two separate traditional selective forces: one selecting for traits themselves, and one selecting for the stabilization of traits, the latter being called “stabilizing” or “canalizing” selection [20,102-104]. The view from my theory is simpler: it holds that there is but one process—that of convergence and stabilization on the population level. There is no need for a separate force of traditional selection for stabilization. Stabilization is an automatic concomitant of the process described here.

## A more detailed look into the new theory

### The writing phenotype evolves, and the writing and performing phenotypes share alleles

I argued that the writing of mutations is an organic process that belongs to the organism. Let us call it henceforth the “writing phenotype”. While traditional theory has had only one kind of phenotype, which we will call here the “performing phenotype”, here we have two: the writing and the performing phenotypes. Let us now derive further theoretical points about how they work.

First, if the writing phenotype is like the performing phenotype, being coded by genes and alleles, then, like the performing phenotype, it must also be evolving.

Second, the writing and the performing phenotypes are obviously different. One implements genetic change, and the other is responsible for survival and reproduction. But although they are different, we can quickly see that they must be sharing alleles, as will now be explained.

As just noted, the writing evolves, and we can now add that it needs to evolve under the influence of natural selection. Otherwise, how could it ever get feedback from the outside world, and how could it be different from random mutation indefinitely, when the performing phenotype clearly changes vastly through the eons in accord with the environment? Without a source of feedback on this outside world and the organism ever-changing with it, mutation must eventually become accidental to the organism. (With no flow of information from B to A, and no predetermination of both B and A by C, A must be random to B).

Now, by definition, the effect of selection is registered in the frequencies of performing alleles. Therefore, if the writing phenotype evolves under the influence of selection, it means that performing alleles influence the writing phenotype. If they influence the writing phenotype they participate in the writing. Therefore, performing alleles are also writing alleles.

There is another way to derive the same point. The writing solves the problem that combinations under selection must have an effect. To solve this problem, it must be that a combination of performing alleles at different loci is taken and an allele is derived from it. This means that performing alleles are inputs into the writing—they affect the writing operation. But if they affect the writing operation, they are writing alleles too.

Thus from both directions we see that the writing phenotype and the performing phenotype share alleles. But the alleles do not mean the same thing to them. The alleles’ full meaning is generated by the way they modify the taxonomically-shared part of the writing phenotype and the taxonomically-shared part of the performing phenotype respectively, which are different.

We obtain the following picture: Alleles participate in the writing of alleles, and alleles are selected. The writing performs an operation, whose inputs are alleles and whose output is an allele. The writing itself always evolves.

This concise statement is what we are led to, and it deserves much reflection. A concrete example will help to explain the idea that the same alleles, and therefore the same genes, can participate pleiotropically in both the writing and performing phenotypes. According to the theory proposed here, the *TRIM5* and *CypA* genes, which participate in the performing phenotype, also participated in complex genetic activity in the germline that eventually led to their fusion [105,106], indeed to their independent fusion in different monkey lineages [105,107-112].

It necessarily follows from the points above that the writing always evolves along with the evolution of the adaptation. It accumulates information from selection, and the alleles that it generates are specific to the evolutionary times. But it is never “ahead of” selection—it never takes upon itself the forbidden role of producing something known in advance to increase fitness—it never replaces natural selection in its role.

We can further illuminate the nature of the writing phenotype by contrasting it with “cranes” [113]. Those are hypothesized phenotypes that are pre-evolved, generic and repetitive devices that supposedly speed up evolution based on a traditional, ns/rm core. An example of a crane is presumably given by the hypothesis that the SOS response system in bacteria induces temporary general hypermutability in response to stress, and that this general hypermutability speeds up ns/rm-based evolution and thus hastens the arrival of a solution at a time of need (reviewed and criticized in [114])^**d**^. “Cranes” such as the hypothesized temporary general hypermutability system would be of long-term evolutionary benefit but are not themselves evolving along with any specific adaptation evolving at present, and therefore are not tailored to any particular adaptation, and still rely on traditional ns/rm to do the “work” of evolving an adaptation. They are thus “add-ons” to the traditional perspective, and they are not easy to justify from that perspective, because the benefit they bring is a long-term, evolutionary one. In contrast, the writing phenotype is not generic. It evolves along with the adaptation. It is therefore specific to the adaptation and the evolutionary times, and only thanks to its evolution the adaptation can evolve. Thus, the theory presented here emphatically agrees with Koonin’s conclusion that evolvability can evolve [115], however, it proposes that evolvability reflects the evolution of the writing phenotype. This is a far more direct explanation for evolvability than high-level selection.

Interestingly, Wagner has already noted that one of the most interesting things that transposable elements demonstrate vividly is that the options available for genetic evolutionary change are specific to the evolutionary times [18]. In a sense, I am generalizing Wagner’s deep insight here from transposable elements to the entire writing phenotype.

We can conclude that the writing phenotype and the performing phenotype evolve together. Indeed, their coevolution explains how they relate to each other syntactically—they never “lose track” of each other. Nonrandom mutation is neither a Lamarckian-transmissionist “seer” that usurps the role of natural selection nor an “add-on” on top of traditional selection. It is a continually evolving system that sits at the heart of the adaptive evolutionary process.

### The new theory predicts that genetic activity implementing the writing of mutations exists in the germline

Several easy predictions now follow from the above.

First, for the writing of mutations to have an evolutionary effect, it obviously needs to take place in the germline. This means that there must be biochemical activity in the germline responsible for the writing of mutation. To continue the example from the previous section, it has been noted that *CypA* is highly expressed in the germline, and that this may have contributed to the independent arising of the *TRIM5–CypA* gene fusion in at least two different monkey lineages [106,116]. While from a traditional perspective we could stop the intellectual inquiry here, and assume that this germline activity is simply an accidental situation, the theory proposed here considers this situation to be the result of a long-term evolution of the writing phenotype, essential for the long-term evolution of the performing phenotype (they coevolve, as stated). In other words, we are dealing here not with accidental boundary conditions, but with evolved writing activity.

Second, according to my theory, alleles from different loci must interact in the determination of mutation. Thus, mutation determination is complex—genes must interact in the germline in the determination of mutation, enabling the fact that alleles interact. The determination of mutations cannot be exclusively simple, single-locus based.

Third, because the performing and the writing phenotypes are different, but they share alleles (meaning, the same genetic difference that plays a role in the performing phenotype also plays a role in the writing phenotype, though this genetic difference has different phenotypic meanings in these two phenotypes), the same alleles will participate in biochemical activities in both germline and soma, but those activities will be different. Hence, genetic activity observed in the germline should not be immediately assumed to be serving the performing phenotype of the germline—it could be writing activity. Furthermore, this activity may involve somatic performance genes.

### Genetic evolutionary trends exist on all timescales

The writing phenotype can be understood better by analogy to the performing phenotype. Four-legged animals use their legs for locomotion by pressing them against the ground. In this general sense, quadrupeds are all similar. But this general description is filled with detail as we move to finer taxonomic levels: horses gallop, rabbits hop. The details continue to be filled as we get to the individual level. Individuals can have shorter or longer limbs, different proportions of fore and hind limbs, different details of their muscular activation, etc. These individual-level details, though small in comparison to the general mode of locomotion, are very important—they are the individual-level variation that is the basis of natural selection. Thus, note that there is a spectrum of contributions to the performing phenotype, including a basis that is persistent and slowly changing, and is generally defined, as well as ever increasing detail that distinguishes between ever finer taxonomic entities and evolves on ever shorter timescales.

Now, I argued that the writing phenotype is an evolving phenotype, and therefore has the same structure as the performing phenotype. In light of the above, this means that there are contributions to the writing phenotype from all taxonomic levels. The more widely shared these contributions are, the more generally they are defined, the slower they change, and the longer the timescale on which they persistently act. Accordingly, at the deep end of this spectrum we find that all organisms have a genetic code, whose characteristics begin to define the range of possible mutations in a very general sense. Further along the spectrum we find that different taxonomic groups have somewhat different methods of gene duplication and different transposable elements, for example, further delimiting the range of possible mutations. And at the far end of this spectrum, writing events in a particular individual are defined in a perfectly concrete manner—these are the particular mutations occurring in the individual. According to the new theory, the details on the individual level are important: they are nonrandom (because mutation is nonrandom), and they enable interaction-based evolution by natural selection.

Note that, whether we take the traditional standpoint or the new standpoint, we must accept that there are ever finer specifications of the range of possible mutations. But while the traditional theory must draw a line at some point and say that “up to this point the machinery defines the range of mutations, and beyond this point mutation is random”, the theory proposed here refuses to draw such a line, and completes the spectrum by saying that mutation is determined by the writing phenotype all the way up to the individual level, and is individual-specific, just like the performing phenotype is. We may call this “individually determined mutation”.

Note also that the line drawn by traditional theory is arbitrary. From a traditional standpoint, we start by assuming that there is a genetic code. Then we add that there is replication or other error, hence point mutation. To account for new genes, needed for the evolution of complexity, it was added that whole gene duplication exists [117]. But now we must assume that we are lucky enough that the genetic system is constructed in such way that gene duplication exists, but that this extraordinarily important machinery of gene duplication [118] must be applied here and there by chance. There is theoretical arbitrariness in saying that, up to here the range of mutation is constrained by the system, and beyond here it is not constrained at all, when no reason is given for why such a dividing line should be placed at one point rather than another. Indeed, the more we study the situation empirically, the more we see finer determination of the range of mutations. Gene duplication is strongly influenced by the location of segmental duplications/low copy repeats (see the section “Evidence from and predictions for molecular evolution”); the location of segmental duplications/low copy repeats is strongly influenced by the location of transposable elements (see the section “Evidence from and predictions for molecular evolution”); and the location of transposable elements is strongly influenced by various sequence characteristics. The dividing line between “mechanistic” and “random” keeps being pushed back. Here I argue that there is no such line. Any line would be arbitrary. The removal of this arbitrary line is an independent point of entry into the new theory, because by removing it, we immediately get to individually determined mutation.

Now consider the existence of the genetic code; the fact that the “error rate” in replication supposed under the random mutation view is not too high and not too low, so that it allowed evolution; the fact that the genetic system is structured such that whole gene duplication, necessary for long-term evolution, is possible, etc. From the traditional perspective, we are lucky that all these things exist, so that evolution as we know it is possible. The existence of these phenomena cannot be easily explained under the traditional theory, because from that theory we normally take them as given and do not begin to think about evolution before we imagine them in place (we do not normally think of them as evolving) (see [57] for an opposing, nontraditional view, consistent with the present work). We cannot say that they are explained by the benefit they bring to evolution in the long term, because traditional theory can only explain the evolution of traits based on short-term, individual-level advantage [6,16]. Indeed, these phenomena are rather parts of the evolutionary “infrastructure”. Since we cannot explain their existence by the traditional process, from the traditional view we can only say that they appeared by chance or by an unknown process outside of the theory. This leaves us with a number of fundamental biological phenomena which enable evolution but are not explained by the traditional evolutionary process.

One possibility is to apply high-level selection to this gross problem [8,119]. However, the whole situation is seen differently from the perspective of the theory presented here. Even though the theory presented here, like the traditional one, cannot explain in detail how these phenomena arose and their current form, the theory presented here inherently includes a mechanism that supports their existence and evolution. Namely, mutations are effected by a writing phenotype. Since this phenotype obeys the same rules of biological structure as the performing phenotype, as explained above, it has long-term enabling effects on evolution (in addition to short-term ones). This succinctly provides a framework for understanding these phenomena’s long-term effect on evolution, which the traditional theory does not. That is, these phenomena define the range of mutations, and are part of the writing phenotype. This framework is entirely different from both sides of the levels-of-selection debate.

An additional, important prediction can now be made. I argued that the more widely-shared aspects of the writing phenotype are more generally defined and more slowly changing, and therefore act more persistently on a longer timescale. If a general writing trait has been in existence for a long period of time, only slowly changing, then it has been guiding the writing activity during that period of time in a somewhat persistent manner, giving rise to some degree of “directionality” in genetic evolution. I predict that this directionality will be observed in the form of hitherto unexplained long-term genetic evolutionary trends. These trends do not define the evolutionary changes completely. They are rather filled with detail at finer taxonomic scales. And although they constitute a certain amount of internal guiding to genetic evolution, this internal guiding does not work by itself, but only together with natural selection, and is in fact itself the result of past selection and writing.

### Context-dependent selection participates in the formation of the phenotypic meaning of an allele

When selection operates on interactions—meaning it is context dependent—then the change in the frequency of an allele is inconsistent in its direction, because this change depends on the context of other alleles, which is itself changing at the same time. The dynamics of allele frequencies are nonlinear.

Context-dependent selection has two interesting consequences. The first concerns the phenotypic meaning of an allele.

In the traditional mindset, we think of effective selection as acting mostly on independent alleles. To be precise, random mutation arises that interacts with the fixed genetic background but not with concomitant alleles at other loci, and in that interaction with the fixed genetic background it has its own phenotypic meaning that is complete at the moment of the arising of this mutation and that is unchanging throughout the period of its selection. All that remains for natural selection to do is to check whether this mutation is “good” or “bad” in and of itself. Thus, in the random mutation case, selection is an external judge of a phenotypic meaning formed at random before selection takes place.

In stark contrast, under context-dependent selection, the phenotypic meaning of a spreading allele (an allele whose frequency is increasing, albeit inconsistently) depends on which other alleles are spreading. But which other alleles are spreading is affected by selection on interactions. Therefore, natural selection affects the phenotypic meaning of an allele—*it participates in forming this meaning*. Thus, according to my theory, selection is not an external judge of a pre-made phenotypic meaning, but is an active participant in the formation of it. This alone means that the phenotypic meaning of a mutation is not random to natural selection, because information from natural selection is already in it. Selection is inside, not outside, the process of formation of the phenotypic meaning of an allele.

At the beginning of this paper we found that the need for selection on interactions to have an effect is answered by the writing of mutations—by genetic change having a mechanistic and organic basis, and in that sense being nonrandom. Now we have just derived from selection on interactions that selection participates in the formation of the phenotypic meaning of an allele, which shows that the phenotypic meaning of genetic change is not random. Interestingly, these two points naturally come together, defining nonrandom mutation from above and below.

### What appears neutral under the assumption of additive alleles can actually experience selection on interactions

The second point of interest that follows from context-dependent selection concerns the neutral theory. Haldane’s [120] calculation of the “cost of natural selection” was an important reason behind the advent of the neutral theory [121]. This calculation had put a severe limit on the rate of substitution that could be driven by traditional natural selection, and the actual rate of substitution [122] as well as the amount of present genetic variation [1,2] later discovered vastly exceeded this expectation [121]. Hence Kimura proposed that the vast majority of mutations are simply not under selection and just drift to either fixation or extinction [121].

However, the theory presented here holds that selection operates on interactions; and since Haldane’s calculation was based on traditional assumptions, here it simply does not apply. Moreover, when selection acts on interactions, alleles exhibit inconsistent change in frequency, which may appear to us as drift. In other words, alleles that appear to be drifting may actually be experiencing selection on interactions. What looks neutral through the lens of the traditional, additive-effect–based theory may not be neutral from a selection-on-interactions view. This does not mean that traditional drift cannot exist in addition to selection on interactions, however, it does suggest that so-called “neutral” matter can be subject to selection and thus has a vast adaptive potential.

## Evidence from and predictions for molecular evolution

We may categorize mutation into two high level categories: rearrangement mutation and point mutation. I will discuss them below in turn.

### Rearrangement mutation is nonrandom

It is now clear that the genome is highly dynamic, involving a great deal of rearrangement—where sequences are duplicated, deleted, inserted, inverted or translocated [17]. This ongoing rearrangement is a new reality in molecular biology—exposed by modern technologies and unknown at the foundation of the evolutionary synthesis. This rearrangement was first thought to be random, but it is now clear that it is locus-specific, that it is effected by biological mechanisms, and that these mechanisms are guided to their places of action by DNA sequences [123-125].

Four main categories of mechanisms of rearrangement are: non-allelic homologous recombination (NAHR), non-homologous end-joining (NHEJ), replication-based mechanisms (RBMs) and transposition.

NAHR [126] occurs when sufficiently long non-allelic sequences of high homology align and cross over. When this crossing over is between homologous chromosomes or sister chromatids, the result is a duplication and/or deletion of the sequences between the non-allelic homologous sequences and of one of the non-allelic sequences. If it is between repeats on the same strand that align as the strand coils, there are two options: if the repeats are in direct orientation, the result is a deletion (transposable elements are often precisely excised in this way [17]); if they are inverted, the result is an inversion. And if this crossing over occurs between repeats on non-homologous chromosomes, the result is a translocation. Notably, duplications, deletions, inversions and translocations of whole genes would not have been possible without a mechanism to enact them, and there is elegance in the mechanism of homology and recombination that is able to produce quite different outcomes based on different parameters of the situation, and that indeed is also the basis of sexual recombination.

The regions of sufficient homology are usually provided by low copy repeats (LCRs) or segmental duplications (SDs)—terms that are used interchangeably, though defined originally independently using somewhat different parameters (SDs were defined as segments ≥ 1 kb in size and ≥ 90% sequence identity [127], and LCRs were defined as intrachromosomal duplications ≥ 10 kb in size and ≥ 97% in sequence identity [128]). It is thought that ∼5% of the human genome consists of LCRs/SDs, and they are particularly prevalent in pericentromeric and subtelomeric regions (reviewed in [129]). NAHR can also occur between tandem duplications, and more rarely between repetitive sequences, which are shorter and much more numerous in comparison to LCRs/SDs (transposable elements constitute about half of the human genome). In this case, the repetitive sequences are expected to be closer to each other as compared to the LCRs/SDs that cause NAHR, and the rearrangements tend to be smaller [123,124].

NAHR is not random. Not only does it require the biological mechanisms of crossing over to be implemented, the LCRs/SDs specify the locations where it usually takes place, and it is often recurrent [123]. Indeed, the breakpoints are further specified within the LCRs/SDs, where they are clustered in narrow hotspots, often nearby DNA sequences such as direct and inverted repeats, which form hairpins, cruciforms and other non-B DNA structures, known to induce double-strand breaks (DSBs) involving enzymatic processes [123,130-132]. Their precise locations can be very close to meiotic recombination hotspots [133], implying the sharing of features with meiotic recombination hotspots [134] (reviewed in [123]), which are known to be associated with consensus sequences and more (reviewed in [135]; to be discussed later). Furthermore, the non-allelic sequences causing NAHR have functional relatedness: they share long sequence homology, and we know that sequence defines function; and the recombining LCRs/SDs need to be sufficiently close to each other (the more so the smaller they are) (reviewed in [123-125]), either by simply being nearby on the chromosome or because the three-dimensional structure of the DNA brings them together from regions that are remote in two dimensions, and we know that closeness in two dimensions as well as in three dimensions is to some degree related to function [125].

Non-homologous end-joining (NHEJ) is able to recognize two ends of DNA (double stranded), modify them and join them together. If the two ends come from two distant points rather than one, a deletion or inversion occurs [136]; and if they come from one point, but double-strand break homologous repair is performed before the end joining, it can lead to duplication [137,138]. NHEJ is nonrandom: it occurs in hotspots (e.g., [137]), though they do not cluster as sharply as in NAHR [123]. These hotspots are often within repetitive elements such as LINE and *Alu* and near sequence motifs that can curve DNA and cause DSBs, and one of the breakpoints in a rearrangement event is often found within a LCR (though the LCR is not necessary for homology in this case) (see [124] and references therein). Thus, local genome architecture influences the occurrence of these events.

Complex rearrangement events by mutational mechanisms are also possible, and replication-based mechanisms (RBMs) have been proposed that may cause such events. In general, RBMs include replication slippage (RS; [139]), serial replication slippage (SRS; [140]), fork stalling and template switching (FoSTeS; [141]) and microhomology-mediated break-induced replication (MMBIR; [142]). In replication slippage [139], microhomology between short repeats allows the nascent strand to move a few base-pairs forward or backward on the template strand and continue replication from there, which causes a short duplication if it moves backward or a short deletion if it moves forward. In serial replication slippage [140], multiple forward and backward movements within a replication fork can occur, leading to a small but complex rearrangement event. Invasion of a new template due to microhomology between inverted repeats can also lead to synthesis of an inverted segment. The FoSTeS [141] and MMBIR [142] models propose that the lagging strand from one replication fork can disengage and invade another fork that is probably close to it in 3-D space based on microhomology and continue replication there, leading to deletions, duplications, inversions and/or translocations based on the parameters of the situation. Serial disengagements and invasions can lead to complex rearrangement events, as in the single-fork case, but this time they involve larger sizes of segments and larger distances between segments. These mechanisms do not act randomly, as they involve microhomology and non-B DNA structures [123,130,143,144]. Clear groupings of breakpoints have been observed in some cases that have been attributed to FoSTeS/MMBIR [141,145]. And closeness in 3-D between forks may suggest related function as mentioned [125]. Although the mechanistic aspects of NHEJ and RBMs have been illuminated by studies of genomic disorders, these mechanisms may account for the majority of non-pathological copy number variation [142,146,147].

Rearrangement by transposition is the fourth main category of rearrangement changes. It occurs when transposable elements (TEs) move themselves as well as other pieces of genetic material incorporated in them—DNA transposons and insertion sequences with the help of the enzyme transposase (with or without maintaining a copy at the source), and retroelements with the help of the enzyme reverse transcriptase and integration into the DNA (always maintaining a copy at the source) [17,148]. As will be discussed later, one school of thought, associated with the traditional framework, has held that TEs are “selfish elements”—parasites of the genome—and that occasionally they are coopted by chance for other functions [5,6,149]. But we will see later how TEs can have the appearance of selfish elements yet be an inherent part of the mutational mechanisms that serve the evolution of the organism. Indeed, they donate every kind of functional element, including promoters, enhancers, splice sites, coding sequences and sequence motifs, and have an extraordinarily wide and deep range of evolutionary influences [15,17,148,150,151]. TE movement is not random. They have a wide range of preferences for target sites, some showing affinity to certain chromosomes, others to loci distinguished by certain sequences, others to loci of a particular nucleotide composition, etc. [17,148]. It is also thought that TEs are involved in the formation of SDs/LCRs discussed before. *Alu*s have been observed at the end-points of nearly 30% of the LCRs/SDs in humans [152,153], implying *Alu*-based homology is involved in their proliferation.

Korbel et al. [146] and Kidd et al. [154] systematically analyzed structural (rearrangement) variation breakpoints in the human genome, and have found that almost all breakpoints analyzed have signatures of one of the four mechanisms above. As previous authors already noted [123,124], this means that the vast majority of rearrangements in humans are due to biological mechanisms whose action is directed by DNA sequence and structure and are therefore not random. We need only to add that this sequence and structure is itself evolving.

### Point mutation is nonrandom

We discussed rearrangement mutation above. The other general category of mutation is point mutation, nowadays referring to a single nucleotide change from one of the four kinds of nucleotide to another. Naturally, we used to think that these changes are random, but cutting edge research in molecular biology is showing that, as in the case of rearrangement mutation, a great deal of point mutation is nonrandom.

Point mutations are not uniformly distributed at random across the genome, but instead the mutation rate per locus varies across the genome on all scales, from the single-base resolution through the gene scale and mega-base scale to the chromosome scale [155].

Many point mutations in humans are due to a change in the cytosine of CpG dinucleotides (dinucleotides where cytosine is adjacent to guanine in the 5’-CpG-3’ orientation) [156] that are spread out over the genome outside of the relatively narrow CpG-rich regions (themselves not experiencing this high rate of mutation; [157-159]). This change is due to methylation of the cytosine, which, in this CpG context, is the predominant target of DNA methylation in vertebrates [159,160]. The methylation is enzymatic and controlled by evolved machinery, and following deamination it leads to a C→T mutation at a very high rate (reviewed in [155]). This high rate of transition is either because of chemical instability of the methylated cytosine, or due to an enzymatic process yet to be discovered [161]. However, we already know that this kind of mutation is nonrandom because of the biological marking of the cytosine, which causes the mutation one way or the other. Notably, ∼24% of all point mutations in humans are due to this mutational process [156].

In addition, ∼18% of the human genome is within 10 bp of a CpG, and an ∼50% increase in single nucleotide polymorphisms (SNPs) has been observed within this distance in methylated regions [162]. It has been proposed that deamination of the methylated cytosine is followed by “error-prone repair” which not only establishes the C→T mutation but also gives rise to point mutations in nearby bases at the same time [162-164] (but of course, “error-prone repair” may also be called a “change-inducing mechanism”).

Other short sequences also exist that have a substantial association with mutation rate. The sequences ATTG and ATAG have a mutation rate of T→C in the second position that is 3.5- and 3.3-fold higher, respectively, than the genome-wide average T→C mutation rate, and ACAA has a mutation rate of A→C in the first position that is 3.4-fold higher than the average A→C mutation rate, in humans [165] (compare to a 5.1-fold excess of C→T mutations in CpGs in these data [165]). The average mutation rates of other short sequences also differ significantly amongst each other, and farther nucleotides also have a significant but ever smaller effect (reviewed in [155]).

In addition, there are loci at the single-base resolution that undergo point mutation preferentially even though no simple sequences have been found yet in these loci [166-168]. We know of these loci from studies of coincident SNPs (cSNPs), where SNPs are observed in the same locations in related species [166-169] (understandably, they also tend to exist in the same loci where single nucleotide substitutions are observed in between-species comparisons; [166-168,170-172]). It has been said that traditional natural selection does not appear to explain these coincidences in the location of variance [168], and so we know that these mutations are guided, though we do not know how. According to Hodgkinson and Eyre-Walker [155], this part of the variance in the human mutation rate across loci that is accounted for by cSNPs unexplained by simple context, called “cryptic variance” [166], is as large as that of CpG mutations (the latter alone involving ∼24% of all point mutations in humans, as said). Thus, we already see that a large percentage of the total variance in the per-locus mutation rate in humans is accounted for by cSNPs and CpG mutations, two obviously nonrandom processes.

It is also worthwhile mentioning that there is a strong association between meiotic recombination hotspots and mutation hotspots [173,174]. Meiotic recombination hotspots move rather quickly during evolution—i.e., they are not conserved between humans and chimpanzees—but they remain within a certain region for longer periods of time (in other words, they move quickly on the single-base scale but more slowly on the Mb scale; [175,176]). Within these regions, substitution rates are elevated [173,176]. Importantly, meiotic recombination hotspots are clearly nonrandom: their locations involve DNA sequence motifs and, according to Wahls and Davidson [135], are determined by the combinatorial effect of the binding of multiple transcription factors at multiple transcription factor binding sites. This complex determination of meiotic recombination locations, interesting in and of itself, will be discussed later, but in the present context it implies that the point mutations co-localized with recombination hotspots are also nonrandom, as their location is biologically determined (even without further direct evidence speaking to these mutations, we know that their rates could not be randomly elevated particularly at those places where recombination is nonrandomly placed).

Finally, as discussed in the previous section, rearrangement mutations are nonrandom, and point mutations and rearrangement mutations are in general related. The rate of point mutation is substantially increased near insertions and deletions (reviewed in [155]).

Taking together the predictive power of simple contexts, of cryptic variance, of the recombination–point mutation association, and of the association between the locations of rearrangement mutations and point mutations, we already know that much of point mutation is nonrandom and under biological control.

### The traditional theory leads to paradoxes when facing new knowledge from molecular biology

Traditionally, we had been thinking that mutation was random and caused by external agents such as UV radiation or toxic chemicals, or by “copying errors”. But we now see that a great extent of genetic evolutionary change is under biological control. Applying traditional thinking to this observation, we still say that all of this mutational activity must ultimately be accidental to the organism: that the biological mechanisms cause it by making errors as they try to restore the previous genetic state or by failing to recognize that state following an accidental disruption. But this view leads to paradoxes.

One such paradox is that mutation hotspots are particularly concentrated in zones of adaptive evolution. This is indicated in several ways. First, genes whose products interact rather directly at the molecular level with the external environment, like chemo-sensory perception genes, immune and host-defense genes, and metabolism and detoxification genes, display a high concentration of mutation hotspots [129,177-179], and to some degree we have independent evolutionary-ecological reasons to expect to see much adaptive evolution in those genes [180]. Second, a high *dN/dS* ratio (a high ratio of non-synonymous substitutions per non-synonymous site to synonymous substitutions per synonymous site) has been observed in such genes [181,182], an observation commonly used as an indicator that genes are under pressure for change. Thus, mutation hotspots are concentrated in zones that, for both reasons just mentioned, are expected to be under pressure for change. Indeed, these mutation hotspots are not just there and disassociated from the adaptive evolution of these genes, but rather appear to play an active role in this adaptive evolution, as demonstrated, for example, by the defensin gene clusters [129]. Third, evidence arising from detailed studies of particular cases, such as evidence of hypermutability in toxin-encoding genes in snails of the genus *Conus*[183,184] and evidence of hypermutability of *HoxA13a* in zebrafish and related taxa (Cypriniformes) [185], is consistent with a connection between mutation hotspot locations and adaptive evolution.

But how did mutation hotspots come to be concentrated where they are needed? The traditional view cannot explain this association well, because this view requires an immediate benefit for the spread of a mutation based on an advantage that it supposedly brings in and of itself, whereas the “benefit” from the presence of these hotspots is due to changes that they bring in the evolutionary long-term, and which are part of the evolution of the population as a whole. What the association rather means is that the biological control of mutations is not incidental to adaptive evolution.

### The association between zones of adaptive evolution and genetic disease can be understood as evolutionary friction points between the writing and selection

It is not the intention of the new theory to suggest that, since mutation hotspots are placed in zones of adaptive evolution, they can “outguess” natural selection. It rather suggests that mutation hotspots are positioned in a long-term evolutionary process that is constantly receiving feedback from natural selection, and they never take on the role of natural selection. This point is underscored by the fact that mutation in mutation hotspots often leads to recurrent genetic disease [186,187].

The importance of recurrent genetic disease has been becoming clear in the last decade, and is a bit of a curiosity from the traditional perspective. In fact, there appears to be a triple association between mutation hotspots, zones of adaptive evolution, and genetic disease (e.g., [129,131,180,181,188,189]). The new theory offers the following view on this situation. Recurrent genetic disease represents evolutionary friction points, where the pressure for change that comes from the writing phenotype and its mutation hotspots—which, according to the new theory, belong to writing mechanisms that have been evolving in the long-term—clashes with the pressure for immediate performance of the focal loci in the context of the current state of the organism. That is, improvement in a complex system is hard to achieve, and it takes an evolutionary “negotiation” process between writing and selection pressures, until either the focal writing trend readjusts and pushes in a new direction, or other loci change and remove the block imposed by natural selection on this writing trend and allow it to persist in its direction. This gives us a way of understanding the triple association just mentioned. In contrast, from the traditional perspective, the long-term persistence in particular places of mutation hotspots that are enabling of adaptive evolution in the long-term yet are costly in terms of recurrent genetic disease in the short-term has no equally intuitive explanation.

We can conclude from the molecular biological evidence so far that the biological control of mutation is plainly fitting with the theory presented here, and in fact connects the two grand phenomena of sex and nonrandom mutation; but it leads to paradoxes from the traditional one.

### The new theory predicts that the determination of mutation is complex, and this prediction is confirmed

Not all of the variance in the mutation rate across loci is predicted by simple context—i.e., by a simple consensus sequence that is present in every locus where the mutation happens. And the presence of one of the simple consensus sequences in some locus does not in and of itself guarantee a mutation in that locus, it only increases the likelihood that we will see a mutation there. As mentioned, a substantial amount of the variance in the mutation rate across loci is cryptic [166].

This is in accordance with the theory proposed here. If all point mutations were completely determined by simple local context, this would not allow alleles from different loci to be involved in the determination of mutations, because each locus in this case would specify its own mutation by itself; whereas, cryptic variance means that mutation is nonrandom, yet local allelic information does not completely determine it, implying that allelic information from other loci participates in its determination, exactly as predicted by the theory proposed here.

Interestingly, Wahls and Davidson [135] argued that simple consensus sequences are not sufficient for the complete determination of meiotic recombination hotspots. Rather, the meiotic recombinational activity is determined combinatorially by the binding of multiple transcription factors that interact with each other [135]. In addition, we know that meiotic recombination hotspots are also mutation hotspots, as said. Combining these two facts, we see that, at least in the case of this type of hotspot, the location of mutation is determined combinatorially by the binding of multiple interacting factors, much like the location of transcription is determined in the performing phenotype (writing function is determined much like performing function). This enables alleles from multiple loci to interact in the writing. Thus, recombination–mutation hotspots as described by Wahls and Davidson are a living example of the individually determined mutation predicted by the new theory—the writing phenotype.

### Evidence of “divergent parallelism” is in accord with the new theory

Cryptic variance relates to another interesting point. In *The Origin of Species *[60], an observation of high generality is emphasized, according to which traits that have been experiencing adaptive evolution in recent evolutionary time are also the ones that continue to vary substantially between individuals at present. It is interesting that this very general observation, so important to Darwin, has not had an obvious place within the traditional (neo-Darwinian) theory: according to traditional theory, which is based on random mutation, variation is supposed to hit where it hits, selection is supposed to act where it acts, and there should be no relation between the two.

Now, molecular evolutionary studies, including those investigating cryptic variance, produce evidence that precisely mirror Darwin’s observation at the molecular level: loci of substitutions between related species (recent evolution) are associated with SNPs (present variance) [166-168,170-172] and with regions of adaptive evolution [182].

From Darwin’s observation alone one could have inferred that there is some long-term persistence in what evolves and therefore some directionality in evolutionary change. Furthermore, since there is persistence in what varies, there is parallelism in what varies, and this parallelism cannot be explained by similarity of selection pressures experienced by related species alone—there must be also similarity in the guiding of the variance. Furthermore, since separate species cannot forever evolve in exactly the same way, but must gradually become more and more different, Darwin’s point implies that evolution proceeds by what might be called “divergent parallelism”: something guides the variance but it gradually evolves. All of this is exactly in accord with the theory proposed here: the writing phenotype evolves.

Indeed, while presenting their results on cryptic variation, Seplyarskiy et al. [168] noted that while a substitution in a certain locus in gorilla increases the chance of an SNP in the corresponding locus in humans by about 30%, the substitutions that have occurred on the path connecting two species of lemurs show practically no correlation with the locations of SNPs in humans. Consistent with this example, they suggested that “[p]erhaps the patterns of the cryptic variation of the mutation rate are subject to evolution and, thus, become more and more different in more and more distant genomes”. This suggestion of divergent parallelism is precisely in accord with the new theory.

### A new interpretation for recent findings

In light of the new theory proposed here, new interpretations become possible for some recently discovered puzzling phenomena. I will discuss under this heading *de novo* genes, epistatic capture, the interpretation of TEs and of junk DNA, transcriptional promiscuity and the unusual genetic activity in sperm cells.

#### De novo gene evolution may be subject to indirect natural selection through the writing phenotype

All would agree that random, accidental mutation cannot be expected to suddenly produce out of thin air a large and complex beneficial change. Therefore, the Fisherian theory of evolution [21], which has been so important in our understanding of adaptive evolution, has a basic idea behind it: it is to minimize the amount of “useful work” that random mutation can supposedly do in any one mutational step. The idea, then, is to let natural selection check each mutation and let through only the useful ones, and thus gradually accumulate the small, additive effects of many such mutations into a substantial phenotypic change [190].

One question that arises, then, is how a new gene emerges. A gene is a complex entity that cannot arise out of thin air. It includes hundreds or thousands of bases of DNA, including both regulatory signals and RNA- or protein-coding sequences, and it cannot be active and subject to traditional natural selection until many of those bases are in place. For this reason, it was rightly suggested already in the 1930s that new genes originate by whole gene duplication [117]: First, a previously complete and active gene is duplicated by a single “duplication mutation” all at once along with its regulatory and coding sequences. Then, point mutations may gradually accumulate in one or both of the copies, eventually making them substantially different from each other and thus leading to the arising of a “new gene” [191]. In line with this, Jacob argued in 1977 that it is impossible to get a gene out of nothing [14]—a gene always starts by drawing on the pre-existing. The word “alchemy” [192] may be attached to this impossibility of complexity out of thin air.

One deep philosophical problem with gene duplication from the traditional theory has already been discussed: it is that we are lucky to have the mechanisms that enable duplication mutations, indeed the mechanisms discussed earlier, because they are necessary for long-term evolution, but their existence is not easily explained by the traditional theory. But there is another problem, raised by recent evidence.

Since 2006, results have accumulated showing the existence of a complete sequence of an active gene in one or a few closely-related species, and the existence of substantially similar (syntenic) sequences in multiple related species that are incomplete and are missing some of the regulatory signals and coding sequences that make the gene what it is in the species where it is active [9-13,193-201]. Because of the nature of the phylogenies involved, it has been inferred (and on this all agree) that the common ancestor of those sequences was nonfunctional (because if it were functional, a larger number of independent evolutionary events of repeated dysfunctionalization in multiple species would need to be assumed); and thus, in the course of evolution—in fact in the course of millions of years of evolution—signals and coding sequence elements have been gradually added until the sequence has become an active gene in one or a few closely related focal species. But this means that, in these cases, referred to as cases of “*de novo*” genes, a gene has been created not from copying and gradual change of a previously complete gene, but in a way that appears, from traditional theory, out of nothing—out of “junk” [192]. That is, multiple random mutations supposedly had accumulated before the gene was activated and thus before they could have experienced traditional natural selection, and these mutations created a whole, functioning gene. It was therefore simply inferred that Jacob was wrong [192], and that random mutations unchecked by natural selection can accumulate and create a whole new gene after all.

I side with Jacob, however, that this should not be possible. But this means that the facts are not fitting with traditional theory^**e**^.

The results from *de novo* gene studies are so tantalizing that they should have received more attention than they did, and that Siepel noted in 2009 [192] that much care should be taken to ensure that they are not due to some methodological fluke.

Assuming that these results hold, the theory presented here can offer an explanation to them, and is the only theory currently offering an explanation: according to it, the writing phenotype can bring information into the evolving *de novo* locus from elsewhere in the genome over the long-term.

According to the theory presented here, the writing of mutation brings together information from multiple loci into the single locus where the mutation is written. So, bringing information into an evolving locus from elsewhere is already part of this theory. Indeed, looking at the data of Levine et al. [193], one can see large insertions in the sequence of the evolving *de novo* gene. Presumably, these insertions have come from elsewhere. In some other studies, *Alu* elements have been observed to contribute to sequence evolution of *de novo* genes (e.g., [12,200]). The involvement in the evolution of *de novo* genes of *Alu*s—the same elements so thoroughly intertwined with the mechanisms of nonrandom mutation discussed earlier—throws much light on the topic, because the involvement of counter-traditional elements joins the impossibility of gene-out-of-nothing in placing the evolution of *de novo* genes far from the reach of traditional theory. In short, I argue that *de novo* gene evolution demonstrates long-term movement of information by writing mechanisms. This is in accord with Jacob’s assertion that a new gene always draws on the preexisting. Indeed, in my theory, every mutation draws on the preexisting.

Importantly, arguing that the long-term action of writing mechanisms gives rise to a new gene does not imply that evolution has “foresight” of the kind long rejected. The writing mechanisms have evolved and keep evolving under the influence of natural selection. They never “guess” what would be beneficial under natural selection. They do not create information out of nowhere but rather process information that is present. Indeed, the long-term trend that culminates in the emergence of a new gene in the *de novo* locus does not work on its own. Rather, it embeds a new gene in the larger genetic network, while changes in other loci make room for this new gene in the network (evolution according to the theory presented here is based on interactions—on network evolution). And, this long-term writing trend itself evolves in the long-term under the influence of natural selection. Thus, I propose that the *de novo* gene, even prior to its transcription and translation, always evolves under the influence of natural selection, but this influence is nontraditional: it accumulates in the long-term through the evolution of the writing mechanism, and is indirect. The crucial difference from traditional theory is this: traditional theory, by lacking the writing phenotype, has no indirect route by which natural selection can influence the evolving *de novo* locus, and thus reaches the paradoxical conclusion that a whole new functional gene evolves absent natural selection.

**Figure 3:**
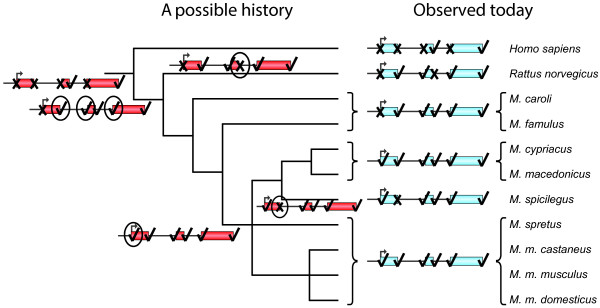
**A schematic diagram showing the evolution of signals in the *Poldi * gene, modified from Heinen et al. **[11]**, with permission from Elsevier.** The visual presentation follows closely that of Heinen et al. [11]. Exons and introns are not drawn to scale. Observed genes are shown in blue, and a possible history, consistent with the nonfunctional-ancestor consensus view in the literature, is shown in red. Checks and crosses represent presence and absence of signals, respectively. According to a parsimony-based interpretation of the data, a possibility arises that signals have been added on the timescale of millions of years. Note that the total number of signals is monotonically increasing with decreasing phylogenetic distance to *Mus musculus* (as the clade including *M. cypriacus*, *M. macedonicus*, and *M. spicilegus* can be rotated around its base).

Armed with this new theoretical framework, we can take a closer look at the *de novo* gene data. Consider, for example, the case of the *Poldi* gene analyzed by Heinen et al. [11]. In the house mouse (*Mus musculus*) and closely related species, this gene is transcribed in postmeiotic cells of the testis and shows evidence of functionality (reduced sperm motility and testis weight in knockout mice). In Figure 3, the signals for *Poldi* transcription and splicing are shown for mammalian species of increasing distance from *Mus musculus*. Notice how in humans (the most distant species from *Mus musculus* in the sample), only 2 out of 6 signals are present. In *Rattus norvegicus*, 4 out of 6 signals are present. In the basal *Mus* species *Mus caroli* and *Mus famulus*, as well as in *Mus spicilegus*, 5 out of 6 signals are present. And in the remaining, focal species of *Mus*, all 6 signals are present. By parsimony, it is assumed that the gene was missing at least one signal at the root of this phylogeny, and that therefore at least one if not more signals were added in time. Looking at this phylogenetic tree without preconceptions, we see the possibility of a slow and tentative construction of a gene over the long-term and therefore in multiple lineages, where in the *Mus* genus it reaches the point of transcription first.

Notice that this evolutionary trend seen in the data takes place on the timescale of millions of years. This is an additional problem for the traditional theory, beyond the problem of gene out of nothing (i.e., beyond the problem of constructive evolution before transcription/translation), because we do not see from the traditional view what would spread this activity out over such a long timescale. But it is fitting with the theory advanced here, which predicts long-term trends in the writing.

Indeed, a bit more can be said about the fit between the theory presented here and events unfolding on the long timescale. The theory presented here is a theory of the evolution of interactions. A new gene does not arise in and of itself as a separate event of traditional separate benefit. It is part of a massive network-level evolution. We may expect that the sequence of changes constructing a new gene will take much time to accumulate, because in parallel to it, vast amounts of changes in the genome are made that allow for and accommodate this gene. Thus, much time is taken by this complex evolutionary work.

This timescales issue is an important general point. New bona fide genes, sufficiently different from other genes, generally arise on the timescale of millions of years, regardless of their mechanism of arising. These mechanisms include not only whole gene duplication and gradual divergence, which may be seen as the gradual arising of a new gene from a traditional perspective, but also chimeric genes and other genes that appear from traditional theory to have arisen by sudden events. Thus, from a traditional perspective, rare events are interspersed with continuous evolution that are not really part of this continuous evolution, and we are lucky to have such events at all because they are crucial for long-term evolution, yet apparently, according to the traditional view, evolution does very well without them in the “in between” periods. The situation is seen differently from the theory presented here, which has not two separate evolutions, one for the short term and one for the long term, but genetic evolutionary trends across the timescales that are complementary and work together in the gradual construction of complex genetic networks.

Two more specific predictions can now be raised. First, if long-term writing mechanisms participate in the creation of *de novo* genes, as stipulated by the present theory, then to some degree there may be molecular parallelism in the establishment of *de novo* genes even before the time that they first become transcribed or translated. Such parallelism, if found, could not be explained from the traditional theory. According to traditional theory, parallelism is due to similar selection pressures across the populations or species concerned. That is, if the same random mutation occurs in each population or species independently by chance, it could be fixed in all due to the similar selection pressure. In *de novo* genes prior to transcription or translation, we have a situation where traditional natural selection cannot yet take place, and parallelism here, if found, would be consistent with the theory proposed here and not with the traditional one.

With regards to parallelism in general, note that even though the theory presented here is thoroughly in agreement with the widely accepted notion that the number of independent evolutionary events should be considered as a cost in the construction of phylogenetic trees, it allows much more molecular parallelism than the traditional theory. This is because, according to the theory proposed here, similar selection pressures as well as similar writing phenotypes in related species support parallelism. However, hypothesized parallelism still needs to go “with the direction of the phylogenetic tree” rather than against it. In other words, independent evolutionary events do not all have the same cost in phylogeny construction but are more likely to occur the closer the species under consideration are, because both the writing phenotype and the performing phenotype (and hence natural selection) are more similar there. Thus, we may expect some molecular parallelism in the construction of *de novo* genes, even though, like traditional theory, we do not expect parallel dysfunctionalization of an originally functional gene.

Second, if the writing of mutations can write regulatory information into the evolving *de novo* locus while using information from elsewhere and thus drive evolution toward a functioning product before transcription and/or translation take place, then we also need to consider the possibility that it can move coding sequence information into the *de novo* locus from elsewhere before transcription and/or translation. The *PIPSL* gene (Akiva et al. 2006)—a new gene transcribed in the testis of humans and chimpanzees [202]—provides an example of the kind of empirical evidence that would be relevant here. Zhang et al. [203] found evidence of strong positive selection (evolution of the amino acid sequence) in *PIPSL* in the lineage leading to humans and chimpanzees, even though the gene appears not to be translated in either species. They furthermore argued against the idea that the gene has been dysfunctionalized and that such dysfunctionalization is the reason it appears not to be translated [203]. But because, from the traditional perspective, one does not expect to see a high *dN/dS* ratio with no translation, the authors proposed that the protein is there and that we just have not found it yet—perhaps it is translated during some brief time-window that has so far escaped observation. While they may be right in saying that there is both a signal of selection and a protein, the theory presented here brings up an additional possibility: that there is a signal of selection yet there is no protein. If the protein is searched for thoroughly and is not found, it would be an intriguing negative finding, because it would be understandable from the new theory but not from the traditional one. That is, although it does not necessarily follow from the new theory that there should be sequences currently undergoing positive evolution that are not yet expressed, because for all we know, the transfer of coding information does not lend itself to a *dN/dS* >1 read, the possibility that it does may be pursued as a potential distinguishing factor between the new theory and the traditional one, in the *PIPSL* gene and in other examples^**f**^.

#### Epistatic capture amplifies the issue of *de novo* genes

*De novo* genes exemplify a problem that goes beyond the genes that have been given this name and that in fact suggests that they do not form a separate category. In recent work, Lynch et al. [15] have shown that a network of more than 1500 genes has been coopted for decidualization of the endometrial stromal cells in placental mammals—a key step in the establishment of pregnancy. Lynch et al. [15] and Lynch et al. [204] have furthermore shown that TEs have made a large contribution to the organization of this network, activating genes that had been previously silent. Furthermore, according to Emera and Wagner [205], it appears that for many genes, with examples both in the endometrial decidualization network and elsewhere [204,205], the insertion of a promoter-carrying TE was not sufficient for the activation of the gene, but rather multiple further modifications were required after the insertion. This point was studied in detail in the case of the decidual prolactin gene, *dPrl*, where modifications after insertion provided multiple transcription factor binding sites that bind factors that interact with each other [206]. Emera and Wagner [206] have called this process “epistatic capture”, a name that underscores the importance of multiple changes acting as a whole and not in a piecemeal fashion.

We can now see the essential similarity between the above and *de novo* genes evolution: in both cases we see that multiple changes are needed before a gene is transcribed and can be subject to traditional natural selection. Indeed, the fact that, in this example, before the gene is transcribed, first it is silenced, then a TE is inserted, and then further modifications occur, such as insertions of additional TEs and point mutations, shows that a whole lot must happen before it can become subjected to traditional natural selection in its new context. This multiplicity of changes is essentially like that of the evolution of *de novo* genes, and expands the point from the *de novo* genes section, because now the multiplicity of changes is thought to happen in each of many genes that are part of a network.

The fact that TEs are involved both in epistatic capture and network organization and in *de novo* gene evolution is of further interest. In accord with the new theory, TEs can participate in bringing information from one locus to another, and, since their movement is affected not only by themselves but also by sequences at or other characteristics of their point of insertion, TE movement in fact combines information from multiple loci as it generates mutation.

Note that in the case of the evolution of endometrium decidualization, as in the case of *de novo* genes, the multistep process takes millions of years. We have already discussed how this spread of activity over the long-term fits with the new theory but is unexplainable from traditional theory.

Lynch et al. [15] have argued that their results demonstrate that novel, complex adaptations evolve not by the traditional process of independently selected steps but rather by network-level evolution. Though they did not propose any new theoretical development in that regard, their statement is thoroughly fitting with the theory presented here, because network-level evolution is interaction-based evolution.

#### Addressing the conflict in the interpretation of TEs

There have been two ways of interpreting TEs. One emphasizes that they are serviceable to the organism [15,19], [150,207-210]. The other sees them as “selfish elements”—parasitic material—“junk” [5,6,149]. According to the latter, TEs are the remnants of viruses that replicate themselves “for their own selfish benefit” at the expense of the “host”, though occasionally, by chance, bits and pieces of them are coopted by the host for host use. This “selfish genes” school of thought has much to commend itself by, because if we focus on the short-term, it looks indeed as though TEs just “replicate themselves” and are not really needed for the organism’s performance; if anything, they can cause disease. The problem is that, while a “little bit” of fortuitous cooption of TEs for “host” use may seem reasonable to assume, the immense contribution of TEs in the long-term to the evolution of organisms is a bit hard to assimilate under traditional principles. Would supposedly fortuitous movements of genomic parasites organize more than 1500 genes into a novel genetic network underlying the important, complex adaptation that is the decidualization of the endometrium [15,204]? The situation from a traditional perspective is a stalemate: both sides have important arguments to support themselves, yet they are conflicting. Doolittle [8,119] offered a way of resolving this conflict, by proposing that clade-level selection helps to explain the existence of a system hospitable to TEs which is useful in the long term. But the debate over whether selection at levels above the gene and individual is strong enough to affect such things is far from resolved. Hence we must admit that the question is open.

The theory presented here sees TEs as a part of the system—as a part of the writing phenotype. Their contribution is systematic, and does not arise as a fluke. This system, however, is not a “homunculus” that outguesses selection. Instead, it is a complex system—an ecology of writing activities. In such a decentralized system, TEs may well appear to be “pressing” to self-replicate and integrate where they can, even while other writing forces remove them, change them, or silence them; and it is through this tension of writing activities that evolution happens, according to the new theory. The same view of “negotiation” between contradictory forces has been applied earlier here to the understanding of the triple association between zones of adaptive evolution, mutation hotspots, and genetic disease^**g**^.

What of the relation of TEs to viruses? At first sight, it might seem to support the selfish elements view: if TEs evolved from viruses (indeed “viruses” as we see them today—parasites of their host, unnecessary to the host and its evolution) then at least originally, their incorporation in the evolution of the host genome has been a fortuitous cooption of parasite parts; and if this happened originally, we might as well assume that it keeps happening. But we do not know that TEs evolved from viruses originally. The perspective given here, which sees TEs as a systematic part of the organism’s genome, encourages us to consider the possibility that at the origin of viruses were elements much more intertwined with the functioning of the organism.

Another point raised by the present theory regarding viruses concerns the question of how they evolve. They seem to be too small to include much if any writing mechanisms. However, much like their performing phenotype is not “their own”, but due to an interaction between them and the host, so too could their writing phenotype be not their own, but due to such interaction. This leads to a certain prediction: that viruses will show specific characteristics of molecular evolution (idiosyncrasies of specific writing mechanisms) that parallel those of their present and past hosts.

#### So-called “junk DNA” may participate in evolution in a nontraditional manner

The sequences from which *de novo* genes arose are called “junk” from a traditional perspective [192], but I argued that writing activity takes place there. Repetitive elements have been said to constitute “junk” [5], but I argued that they are part of the writing machinery. Thus, according to the theory proposed here, such so-called “junk DNA” may participate in evolution in a nontraditional manner.

Recently, the ENCODE consortium announced, based on empirical evidence, that as much as ∼80% of the human genome is biochemically active and therefore “functional”. This statement was criticized on theoretical grounds in papers that were invaluable for bringing evolutionary theory to bear on the results [7,8]. However, since these theoretical grounds trace themselves back to traditional theoretical assumptions, we must go over the assumptions underlying the criticism and see how they may change once we take the point of view proposed here.

First, it has been thought that junk DNA is junk because it is not conserved. Evolution in it appears to be neutral, and if it were functional according to the traditional theory, it would have represented far too much genetic load [4,7]. However, I have argued that, in accord with the new theory, things can evolve that have a neutral appearance yet are under the influence of natural selection on interactions. Evolution of interactions is explicitly nontraditional, and traditional load calculations do not apply to it. Thus, according to the new theory, these traditional reasons to believe that the majority of the human genome is “junk” do not apply.

Second, it has been thought that junk DNA is junk because of the C-value paradox: organisms may vary greatly in genome size without relation to apparent organismal complexity, which has been taken to suggest that much of the genome is not needed [8]. But first of all, in what sense is it “not needed?” The traditional theory considers only the performing phenotype. Once we admit the existence of the writing phenotype, there is room for much writing activity across the genome. As Eddy wrote, it is one question how much DNA it takes to design a human; it is a whole other question how much DNA it takes to evolve a human [211] (and one may add: the amount of DNA participating in mutational writing in a given organism at a given point in time may not necessarily follow organismal complexity closely).

If junk DNA is part of the evolving writing ecology, then we see that it could vary much and yet be an inherent part of the evolving organism. Indeed, TE bursts and whole genome duplication are obviously of major importance for evolution in the long term, and they cause quick changes in genome size. Those who support the junk concept do not contest this last fact that junk can become of use in the long-term [7,8]; indeed, some have criticized the use of the word “junk” because of this [150]. The issue is rather how we conceptualize the evolutionary process. The traditional view has a difficulty in seeing long-term activity as systematic. TEs serve as an example: their short-term costs have been part of seeing them as “selfish elements”, and their long-term benefit has been seen as a fluke. Thus, the traditional view sees activity in the “junk” as random noise out of which adaptive material arises fortuitously. However, my theory allows for biological activity of long-term constructive consequence even with short-term costs. More generally, junk DNA may play an important, nonrandom role in evolution in the long-term. What we are observing in the non-conserved DNA may be an unconcentrated mass of interacting material out of which more concentrated, “genic” material gradually arises in the long-term by a nontraditional evolutionary process.

Thus, while findings of the ENCODE consortium have already shown that the majority of the human genome is biochemically active [3], the new theory allows for the possibility that this activity is part of the evolving organism and is important; whereas traditional theory seems to predict that only a small amount of it could be functionally important, and the rest must somehow be random noise. The only reason to believe that so much activity in the organism does not really belong to the organism and is just “noise” is the fact that the traditional theory is short-term performance focused, single-allele focused, and random-mutation based. If this reason is removed, our understanding of junk DNA is changed.

#### Many writing mechanisms may exist in the sperm cells

There are several fundamental differences between the genetic system of the sperm and that of the soma. These include, but are not limited to, transcriptional promiscuity (reviewed in [212]), alternative splicing promiscuity [213,214], and a specialized RNA interference and chromatin organization system in the sperm cells of mammals (based on PIWI-domain proteins) [215].

Transcriptional promiscuity (TP) occurs during the development of the sperm cells—in cells at the meiotic stage (spermatocytes) and early haploid sperm cells (round spermatids) [212]. These cells are much more transcriptionally active than somatic cells by several measures: expression is extremely diverse, showing gene products that are different than the usual ones, including partial products, and many RNAs are expressed at much higher levels than usual [212]. TP is a highly involved mechanism, requiring the orchestration of a complex machinery to both create and compensate for the pattern of expression [212], and its evolutionary origin is a mystery.

Interestingly, TP has characteristics that make it useful for the writing of mutations predicted by the theory proposed here [151]. This writing requires bringing together information from multiple loci, which means that at least some genes that affect the writing must be transcribed, so that their products can reach elsewhere and allow interaction between loci. TP can allow many genes to interact and be part of the writing activity, including genes with established somatic functions as well as genes that are in the process of formation and have only minimal promoters [212,216]. The TP stages of the developing sperm cell overlap with the meiotic stage.

With regards to the sperm-specialized RNAi system in mammals, this system is thought to be involved in the control of TEs in the germline [215]. We have already seen the counter-traditional nature of TEs’ role in evolution: they are important in mutational mechanisms and in such examples of evolution as the organization of genetic networks and the long-term writing of *de novo* genes—activities which must be taking place in the germline. It has also been proposed that the germline provides special opportunities for the activity of TEs, especially the state of hypomethylation thought to be involved also in TP [216,217]. It has also been proposed that in mammals there is controlled DNA “repair” by transposons [215], which of course may also be DNA “change”. The existence of such a deeply evolved system that does not abolish TEs but in fact regulates their activity [19,209,210,215] is consistent with the view that TEs are not incidental remnants of viruses that the organism just defends against and that occasionally contribute to its evolution as a fluke, but rather are a systematic and inherent part of the writing machinery.

From a traditional standpoint, one could have raised the question of why all these phenomena with a deep potential to affect the mechanics of evolution occur in the germline. Of course, the germline is where mutations are heritable, and so only there is the evolutionary potential of these highly involved phenomena fulfilled. But from the traditional theory, which is based on random mutation and immediate advantages, one cannot easily explain the evolution of the abovementioned phenomena based on their effect on evolution, and they cannot be easily put together into a systematic picture. The theory proposed here, on the other hand, predicts that the writing of mutations exists, and operates on all timescales. If so, we should expect to see it in the germline. This offers an explanation for the multiplicity of fundamental differences between the genetics of germline and soma.

However, this does not yet clarify all aspects of the situation. My theory predicts that there are writing mechanisms both in the sperm and in the egg (or the cells that lead to them), because both male and female traits must be under the influence of natural selection (and according to my theory, nonrandom mutation collects the effect of natural selection on genetic combinations). Why the asymmetry between sperm and egg in many organisms studied so far? It may be that writing mechanisms that operate in the long-term and evolve in the long-term and that are complemented by individually determined mutation mechanisms, but need not co-occur with them, are free to be either in the sperm or in the egg or in the cells that give rise to them (whereas individually determined mutation must be in both). Thus, if the abovementioned phenomena are writing mechanisms, it means that, for some reason, at least in the organisms studied so far, the developing sperm cells have been specialized as the locus of much long-term writing activity. In this case, why it is the sperm and not the egg cells that have been specialized for this part of the writing is not specified by the theory, but one may wonder if the excessive number of sperm compared to the number of eggs enables this excessive writing activity, for example by providing enough cells so that some will be functional despite intensive evolutionary work causing a high error rate. We may call this “the working sperm” hypothesis.

Indeed, it is interesting that the results of ENCODE, which found so much biochemical activity across the genome, were based mostly on pluripotent stem cells and cancer cells [7], which are known to have unusual genetic activity [7]; according to some, the latter behave in several ways like germ cells, and especially like sperm [218,219]. ENCODE may have helped to bring to light the exceptional genetic activity of the germline.

It is also interesting that new genes have a strong bias of being expressed in the testis, whereas older genes have stronger and broader expression patterns [193,216,220,221]. To explain this bias, it has been suggested that new genes arise first in the sperm to serve sperm functions, and that later these genes are somehow coopted for somatic functions [216,221,222]. This has been called the “out of testis” hypothesis [216,221,222]. Two reasons have been given for why this happens in the sperm specifically: One is the existence of TP there. Interestingly, TP is used in this hypothesis in a manner not far from the above—it is seen there as a phenomenon that facilitates genetic evolutionary change—though its origin in the first place is not easily explained from this view. Second, it was assumed that the sperm are under much stronger pressure to evolve rapidly than other cells in the body, and are thus in “high demand” of new genes [212,216].

However, a main reason to think that these new genes really serve the sperm in its performing phenotype is that knockout of them disrupts the development of sperm. According to the traditional theory, which has only the performing phenotype, this evidence of functionality from knockout indeed means that these genes serve the sperm. But the theory proposed here predicts the existence of the writing phenotype, which raises the possibility that many (though not all) of what we think of as “new sperm genes” serving the sperm’s performing phenotype are not necessarily traditional “sperm genes” but are either genes with an evolving somatic function that are the locus of much writing activity or genes that belong to the body of the writing phenotype, and that disruption of them by knockout derails the writing system and makes it cause damage in the sperm cell (indeed, the sensitivity of sperm cells is well known). Thus, the observation that has led to the assumption that the sperm are under pressure for rapid evolutionary change, which has underlain the out-of-testis hypothesis in the first place, may not be only due to rapid evolution of the sperm performing phenotype. It could be that, to a notable degree, the sperm appears to be so rapidly evolving because of the writing activity in it and the evolution of this activity.

Indeed, we may now note that, of the examples of adaptive evolution detected by *dN/dS* >1 mentioned in [223], the first two (and in that sense prominent) categories of examples mentioned involve molecular environment interaction genes and rapid evolution of sperm, and both of these take on entirely new meaning according to the theory proposed here.

Though much more data are needed on the material discussed in this section, one thing we need to notice is that the “working sperm” hypothesis has relevance beyond science. In 2001, Old [218] (see also [219]) suggested that cancer cells imitate germ cells and trophoblasts in several respects which appear to be part of the malignancy of the disease, including global hypomethylation, expression of cancer-testis (CT) antigens, expression of chorionic gonadotropin, downregulation of the major histocompatibility complex and immune evasion, and more. Indeed, cancer cells are exceptionally genetically active. We should ask, therefore, whether activation of the evolutionary writing activity is part of the etiology of cancer. (It is understandable, then, that in some sense HeLa cells appear “nonhuman” [7,224].)

### A quintessential example of ns/rm may be an example of mutational writing

Since the works of Pauling et al. [225] and Ingram [226], the evolution of malaria resistance and sickle cell anemia has been taken as a quintessential example of the ns/rm process. It has been thought that random mutation caused a change at nucleotide 2 in codon 6 in the *β*-globin gene from A to T (Glu→Val), and that natural selection caused a substantial increase in frequency of this new allele. In the heterozygote form, this allele (henceforth HbS) provides notable protection against the malaria parasite *Plasmodium falciparum*, and in the homozygote form it causes sickle cell anemia [227,228]. According to Haldane’s model of heterozygote advantage [229], selection in malaria-stricken areas maintains the HbS allele in the population despite its cost.

How do we deal with this “queen of examples” of ns/rm from the perspective of the present theory? There are at least two options. First, one may admit that this indeed is a case of evolution by ns/rm. If so, then, if the present theory is correct, one would have to say that this is the exception rather than the rule. In this case, one would say that, occasionally, a random mutation can cause a simple adaptive change, and that the HbS mutation is such a change, but that such mutations cannot build up toward a complex adaptation and therefore cannot be the main drivers of evolution. Essentially this approach was taken by Behe while criticizing the traditional theory [230].

However, there is another possibility. It is that the HbS mutation was not random after all. Consider more background first. HbS is one of several hemoglobinopathies that provide some degree or another of protection against malaria and that are due to genetic changes in the *α*- and *β*-globin genes. The most common of these are the regulatory changes (including whole gene deletion) causing *α*-thalassemia and *β*-thalassemia and the following point mutations causing structural change in the hemoglobin protein: HbS, HbC (which, like HbS, involves a point mutation in codon 6 of *β*-globin, this time causing a Glu →Lys change) and HbE (codon 26 of *β*-globin, Glu→Lys) [231]. Details of the mechanism of protection are still debated [232], but, at the most general level, notice that all these changes affect the internal composition and behavior of red blood cells, which is where the malaria parasite grows. We know that natural selection is involved in their prevalence, mainly from the fact that there is a strong geographical correlation between their prevalence and the incidence of malaria (reviewed in [231,233]). This is consistent with both the new theory and the traditional theory, because both rely on natural selection for the fit between the organism and its environment. It remains to be asked whether the mutations arise randomly or not.

Now, notice that there is substantial mutation and recombination hotspot activity in the *α*- and *β*-globin gene clusters [234,236]. Indeed, some malaria-stricken populations are so riddled with mutations affecting red blood cells that in most individuals the cells are abnormal [237]. From the traditional standpoint, why would these mutation hotspots be there? One might say that mutation hotspots are randomly positioned throughout the genome, and that it so happens that genes with a potential to be involved in malaria resistance also have them. But we have already seen that mutation hotpots are in general frequent in regions undergoing adaptive evolution and are also in general associated with disease. The case of hemoglobinopathies is not a special case but follows this rule.

Indeed, we already know that deletions in the *α*-globin gene, which provide some protection against malaria and cause *α*-thalassemia, occur by NAHR and are recurrent [234]. HbD-Punjab and HbO-Arab, which are the 4^th^ and 5^th^ most prevalent point mutations causing structural change in the hemoglobin protein respectively, are both in the same position—the first nucleotide of codon 121 of *β*-globin, which is right at the sharp bend of a 4-nucleotide hairpin, a DNA structure thought to facilitate mutations [235]. These cases are already consistent with the writing of mutations according to my theory, because they are evidently guided by internal factors. It is only reasonable to ask whether the HbS mutation was also guided by a mechanism yet to be determined.

Indeed, the HbS mutation appears in Africa on four different genetic backgrounds unrelated to each other by simple meiotic recombination events, whereas the HbE mutation appears in south-east Asia on at least two such backgrounds (reviewed in [231]). But HbS does not appear in south-east Asia, and HbE does not appear in Africa [231]. It is usually concluded from these data that HbS arose four times independently in Africa, and HbE arose twice independently in south-east Asia [231]. Now, since there is no reason to expect that these mutations would work in one place but not the other, if these were random mutations, and the rate of random mutation was high enough that HbS arose independently four times in Africa, then why does this mutation not appear in south-east Asia? And if it was high enough so that HbE arose in south-east Asia twice independently, why does that mutation not appear in Africa? To address this question, Flint et al. [231,233] proposed that the HbS mutation arose only once in Africa, and was transferred to other genetic backgrounds by means of gene conversion of short stretches assisted by a meiotic recombination hotspot (which, from a traditional perspective, just happens to be there). But there are multiple other cases of globin gene mutations that show the same kind of pattern, and it is unlikely that this hypothesis applies to all of them (indeed, Flint et al. do not attempt a general explanation, and others do not think that conversion really applies to HbS [234]). For example, according to Flint et al. [231], the *α*-thalassemia deletion mutation -*α*^4.2^ appears to have arisen six times independently in Melanesia specifically, the -*α*^3.7III^ mutation has been found in other parts of Oceania exclusively, and the -*α*^3.7I^ arose three times independently and is most common in the Mediterranean and in Africa. According to Kazazian and Boehm [238], the G→C mutation at intervening sequence 1 nucleotide 5 of the *β*-globin gene (IVS-1 nt 5) appears on different backgrounds in Asia and Melanesia, whereas the G→A mutation at intervening sequence 2 nucleotide 1 of the *β*-globin gene (IVS-2 nt 1) appears on different backgrounds in people of African and Mediterranean origins. According to Thein et al. [239] and Kazazian et al. [240], multiple rare dominant *β*-thalassemia mutations in exon 3 of the *β*-globin gene have been observed outside of the malaria belt in Northern Europeans, notable among them is the third and last possible mutation at the first position of codon 121, G→T [241], which has arisen recurrently there [235], and is different than the abovementioned HbD-Punjab and HbO-Arab, which are mutations from G to C and from G to A respectively, common in other populations. Details may change based on newer information, but the principle is unlikely to disappear. In general, it appears that malaria resistance solutions have a strong tendency to evolve recurrently, and furthermore they are more similar within human populations than between them (this well-established ethnic effect occurs even between separate but geographically neighboring populations; reviewed in [231]).

This ethnic effect of malaria resistance mutations, whose paradoxical nature from a traditional standpoint was well articulated by Flint et al. [231], becomes much more understandable from the theory presented here. According to this theory, the writing phenotype evolves, and therefore different populations will reach somewhat different solutions to the same problem, appearing as recurrent mutation. It is nothing but an example of divergent parallelism, which is how evolution in general unfolds according to this theory.

It would be ironic if HbS—the first mutation to be characterized at the molecular level, and a prime example of traditional ns/rm—turns out to be an example of nonrandom mutation after all. Regardless, in accord with both evidence of other malaria resistance mutations and previous molecular biological evidence discussed, we are left to conclude that, empirically, mutation is affected by internal biological factors, and that these factors themselves evolve. This grand empirical fact is clearly in accord with the theory presented here, but plays no role in the traditional theory and is not understandable in a straightforward fashion from the traditional perspective.

## Final remarks and summary

### The origin of life

It is not my purpose here to make speculations on the origin of life. But without having clarified my position on the origin of sex, it may have been difficult to see how sex could be a matter of necessity for evolution. Similarly, without clarifying my position on the question of the origin of life, it may be difficult to accept the fact that nonrandom mutation is at the basis of evolution.

Let us see, first, that the two origin questions are related. I argued that sex is a matter of necessity. This raised a question of origins: if life began asexually, then biological evolution in the beginning occurred without sex, but this would seem to suggest that sex is not a matter of necessity for evolution. I then argued that, for all we know, life did not begin asexually. But if life did not begin asexually, then it did not start with a single organism either.

People often imagine that life began with a chance event giving rise to a “self-replicating” molecule [149,242,243]. This self-replicating molecule was the “first organism”, so to speak. By self-replicating, it created a population of such molecules, which were then able to compete. Under the assumption of errors in self-replication, neo-Darwinian evolution started and gave rise to all of life, supposedly.

This single molecule/“naked gene” [243] scenario is an extension of the neo-Darwinian idea to the “beginning” of life. Since we must begin with this molecule and end with humans, we then assume that organization at different scales has been added as layers one by one: first came self-replicating molecules, then cells, then multicellular organisms, and then societies.

This view is not the only view on the origin of life (see, for example, [244]), but it serves as a contrast to the theory presented here. If life began with a molecule that arose by chance and self-replicated with error, then life began with a period where there was no writing of mutations—there was no “higher level phenotype” to speak of to enact any change to the presumed chance-arising “genome”. To presume that life began with an asexual, random mutation evolutionary process, and at some point switched to a thoroughly different process based on sex and nonrandom mutation introduces an arbitrary line into history and into theory of the kind that we should be happy to remove and that shows a disagreement between this hypothetical origin scenario and the theory presented here.

But life did not have to start with a single, chance event at some particular point in space and time. Instead, consider the possibility that there has been a smooth transition from a “chemical” primordial soup to a “biological” one. In this case, life “began” with a “proto ecology”—a complex world of chemical reactions. In fact it is not correct to use the word “beginning” here, because it did not start from a particular point in space and time. What would later become the genome and what would later become the phenotype, including the writing of mutations and every other fundamental aspect of life, have descended from this proto-ecology together, coevolving. Genotype and phenotype coevolved. This enables gradual evolution of the framework of life as we see it today, while avoiding sharp transitions from “chemical” to “biological”, from “asexual” to “sexual”, from “single organism” to many, etc. This view is consistent in important respects with those of Woese [54], Brosius [55,56], Vetsigian et al. [57] and others.

One may ask: But did life not have to begin with a self-replicating mechanism, the arising of which has to be explained by a chance event, because without it, there could be no population of individuals to undergo selection? The answer is no. Things can be similar not because their ancestor arose in a point and led to them by asexual reproduction, but because of the application of the same laws and materials everywhere [20]. Complex organismal entities that are similar to each other could have gradually arisen not because of an asexual spread of a chance gene from one point to the rest, but by a process of convergence like the one discussed in this paper. In other words, things can come to share characteristics by multidimensional exchange of information (such an exchange is the shuffling of genes of today), and not just by sequential, one-way spread of non-interacting pieces of information [54-57].

Indeed, “self-replication” is a misleading term. Strictly speaking, there is no such thing as “self-replication”. Do we mean by it that nothing other than the object itself takes part in the replication of the object? This is logically impossible and empirically evident not to exist. An individual can only be replicated in the right environment, not to mention that its replication involves material and energy coming from the environment. Since the right environment is indispensable, the responsibility for replication is not only within the “self”. Furthermore, under sexual reproduction, the individual is not really “replicated” at all.

We tend to put “self” in “self-replication” because the individuals that we see struggle so much to ensure their reproduction, and their teleological behavior makes us focus on them as actors, and because they carry in them information needed for their reproduction. But all of this could have gradually evolved from a “proto-ecology” of chemical interactions, involving the ancestral matter of both writing and performing. Life did not have to start, anthropomorphically, with a chance-like event of the sudden origin of a mechanism capable of “replicating itself”. Life did not have to start with an “Adam” molecule.

### Placing the theory presented here in context of previous thought

When Darwin began thinking about the mechanism of evolution he started by speculating that sex was the driver of it [245], showing how important this phenomenon is in the eyes of the uninitiated. When he saw Malthus’s paper and came up with the idea of natural selection, he largely put the question of sex aside, though he kept in mind a fuzzy notion of “blending inheritance” that is due to sex. When his theory of natural selection became known in 1859, it was not immediately accepted by the biological community (only the fact that evolution happened was), but rather continued to be debated for 70 years, because inheritance was critical to the theory, yet Darwin had only a vague notion of it. Especially, Galton posited that the ever-present individual variation that Darwin relied on could not be the source for evolution, because under blending inheritance—which is due to sex—the individual makeup could not persist; and thus a special, sudden mutation was needed, whose character would not be lost in the passage from one generation to the next [246,247]. We can easily see that, from the beginning, the problem of inheritance was thoroughly intertwined with the problem of sex. It is only since the modern synthesis that we have forgotten that these problems are one and the same. The theory presented here, however, treats them as one and the same: information from allele combinations is inherited through nonrandom mutation, which solves the original problem of sex.

It was not until the works of Fisher, Wright and Haldane in the 1920s and 1930s that Darwin’s theory of natural selection was finally accepted, but only after having been crucially changed. The simplest and clearest was Fisher’s theory of adaptive evolution, and its critical assumption was that each allele is thought to make a small, separate, additive contribution to fitness, or to some quantitative phenotype, and is largely independent of all other alleles. This way, each allele maintains its meaning despite the sexual shuffling, and there is no longer a problem of sexual inheritance (while Fisher acknowledged the presence of interactions, they were not part of the core of his theory of adaptive evolution). It has often been said that the key of the modern-synthetic revolution was that it married Darwin’s theory of natural selection with Mendelian genetics, or in other words with the sexual shuffling of genes. But in fact it proposed a concept of selection that worked despite this form of inheritance, not with it, as until today it is easier to understand this theory without sex than with it.

There is, however, a flaw in Fisher’s theoretical assumption that additive effects are at the core of adaptive evolution. All would agree that an allele does not work on its own as though it exists in a vacuum. The allele must interact with the genetic background. According to the assumption just mentioned, it interacts largely with the fixed genetic background. That is, alleles are not allowed to interact substantially with each other *in general*, because such interaction is “noise” that drowns the supposed signal of single-allele–based selection. But this assumption introduces an arbitrary line into the foundations of the traditional theory: How can we expect that alleles will systematically be able to “distinguish” between genetic partners that are fixed in the population and partners that are not fixed, so that interactions with the fixed background are systematically strong and interactions with concomitant variance are systematically weak? It is interesting that we have not asked this question for so long, but it is time to ask it. Removing this arbitrary line we immediately get to the framework of the theory presented here. This is a third, independent entry point into my theory (independent because removal of arbitrariness from theory is good independent of anything else).

Interestingly, Mayr has already made his criticism of the Fisherian, generally non-interactive approach loud and clear [248]. He considered it of vast importance that selection in the presence of sex is context-dependent, and that, with it, the genome evolves as a unified, cohesive whole. However, he did not propose what makes this context-dependent selection possible—what allows selection on interactions to drive evolution. Here, I have done so.

Finally, Shapiro deserves much credit for being the first to promote heavily an idea of nonrandom mutation. Indeed, he has summarized his view in a highly inspiring and informative book [148]. However, he has not specified the mechanistic underpinnings of the joint operation of natural selection and nonrandom mutation. Also noteworthy and directly relevant to my work are works by Caporale, who has expressed views related to a part of the present paper, namely an association between mutation hotspots and regions of adaptive evolution, which she calls a “mutation phenotype” [177,178]; and works by Stoltzfus and colleagues, who have written extensively on the importance of mutation in evolution from nontraditional views, including on mutation bias [249-252].

### Summary

This paper holds that the mutation that drives evolution is not a result of random accident but an outcome of a mutational writing phenotype. This phenotype itself evolves, like anything else inherent to the organism. It absorbs the information that comes from selection and guides selection further by providing further variation.

I have presented evidence—from Darwin’s organismal-level observations of the evolution of variation, to modern research on cSNPs, to observations collected here on the evolution of malaria resistance genes—that is substantially fitting with the above. Evidence shows that the generation of variance is more similar between more related species or populations, supporting the idea of “divergent parallelism”, which follows from an evolving mutational writing phenotype.

To clarify, this process is not Lamarckian. It is not subsumed by previous discussions of “directed” or “adaptive” mutation (e.g., [22,253-255]) and it would be incorrect to equate it with those (though interesting connections may exist). The nonrandom mutation proposed here does not usurp the role of selection, it rather absorbs information from selection on interactions. This mutation is dependent on the genetic state of the organism, which itself depends on past selection (and past mutations). Importantly, while this mutation is distinctly different evolutionarily from accidental mutation, this does not imply that a given mutation is more likely to arise in an environment where it increases fitness than in an environment where it does not, nor that mutation is more likely to increase rather than decrease fitness.

This process is also not equivalent to “cranes” such as the hypothesized SOS system in bacteria, which are hypothesized generic “tricks” that are presumed to facilitate evolution based on a presumed ns/rm core and that are presumed to have evolved by ns/rm. Quite in contrast, the mutational writing phenotype discussed here evolves along with the evolution of adaptation and is therefore specific to the evolutionary times, and is part of the core of the evolutionary process, not an “add-on” on top of it. In designing experimental evolution approaches in accord with the process described here, one should be mindful of the fact that this process is expected to be generally a long-term one.

The process described here is “Kantian” in that it shows that evolution is driven not only by external forces. It is not random accident that generates the variance that selection operates on. Rather, a phenotype causing syntactic internal change is absorbing information from the outside world—from natural selection—and changes itself in the process.

This solves the problem of sex in a manner very different from before. No longer do we treat sex as a phenomenon of potential subsidiary benefit, but rather we treat it as a fundamental part of evolution by natural selection. The theory proposed here does so by tying sex to the question of interactions. Investigators have suspected from the beginning that interactions must somehow be formed to allow for the evolution of complex adaptation. But how are they formed under selection? Some resorted to chance to explain interactions; but this approach was not followed here. In the presence of transmission of hereditary information through syntactic mutational writing, selection on interactions influences future generations.

To reiterate, mutation combines information from multiple loci as it changes a certain locus. While natural selection operates on the genetic combinations created by sex, the writing of mutations combines information from multiple loci into new mutations, which are not themselves broken by the sexual shuffling, and thus allows the combinations to have hereditary effects according to their fitness. Thus, natural selection and nonrandom mutation work together, where nonrandom mutation allows selection to operate on the organism as a complex, interacting whole. Here, a new mode of information transmission was proposed.

Interestingly, this opens up neutral evolution to a whole new interpretation. With selection on interactions, we no longer expect that each allele will be either neutral or have a tendency to move straight toward fixation or straight toward extinction, but rather the frequencies of alleles will change in an unpredictable manner, owing to the nonlinear dynamics of selection on interactions, and this movement is not necessarily easily distinguishable from drift. Therefore, what has been seen before through a traditional lens as neutral matter can be experiencing selection on interactions and thus can play a non-fortuitous role in adaptive evolution.

Cutting-edge evidence from molecular evolution supports the proposition that mutation is nonrandom. More specifically, evidence on cryptic variance and the complex determination of mutation-recombination hotspots supports the proposition that mutation combines information from multiple loci into one. Many other cases that speak to this latter point may be lurking in the literature and still others may have yet to be empirically discovered. Epigenetic inheritance may follow this pattern of combining information from multiple loci into one, and the whole connection between epigenetic inheritance and long-term genetic changes is a massive area that needs to be explored from the perspective of the present theory.

Another point of interest is how an adaptation comes to be shared among the members of a species. A new trait comes into being not by the sequential spread of mutations that supposedly bring separate phenotypic changes from the individuals in which they arose to the whole population. Instead, while alleles spread, they interact, and the new trait arises at the level of the population as a whole from these interactions. We saw that this is necessarily a process of convergence, where gradually the trait becomes less influenced by the sexual shuffling of genes and thus more uniform across individuals. It is therefore a process of stabilization, one that is an automatic concomitant of the adaptive evolutionary process described here, and does not require an extra traditional selective force specifically for stabilization, as assumed in theories of stabilization or canalization. The writing of mutations enables this process of convergence by combining information from different individuals (and from different loci) over the generations. Interestingly, this convergence process connects molecular evolution to phenotypic-level evolution better than before, because empirically, the evolution of complex adaptation looks like a process of convergence at the population level.

Below is an outline of the main points made in this paper:

1. Mutation is the outcome of a nonrandom, biological process.

2. It follows that mutation combines information from multiple loci into one.

3. By combining information from multiple loci into one, mutation allows selection on genetic interactions to have a hereditary effect according to fitness.

4. This revises the connection between selection on the phenotype and evolution of the genotype proposed in the 1920s–30s in a way that connects the theory of evolution better to modern evidence. Mutation has a complex genetic component, and the causes of variance and the nature of inheritance are not separate issues.

5. This view is a third way of thinking about evolution: it is neither neo-Darwinian nor Lamarckian.

6. Given that selection can operate directly on genetic interactions, sex becomes an element of fundamental importance for evolution, not one of subsidiary, circumscribed benefits, since it is the generator of genetic combinations.

7. It follows from the above that: a) sex is original—it did not evolve from asex; b) sex (or a mix of sexual and asexual reproduction in a species) is not actively maintained against obligate asex, but rather, and more simply, long-term adaptive evolution is not available for obligate asex to arise by.

8. It is therefore predicted that obligate asex arises by breakage and that no fine-tuned adaptations ensuring obligate asexuality exist. This prediction offers a new look into a key open question in plant mating systems, namely why pure asexuals are exceptionally rare. It is confirmed in vertebrate unisexual animals and in androdioecious animals, and remains to be tested in complete cleistogamous species and/or other cases.

9. It is also predicted that putative ancient asexuals have not substantially evolved and diversified in an asexual state. This prediction is confirmed in the case of the bdelloid rotifers according to statements by Meselson, and remains to be tested more thoroughly in these organisms and others.

10. It follows from the theory that an adaptation arises by a process of convergence as defined in this paper, and not by the accumulation of separate effects. It arises at the level of the population as a whole from genetic interactions.

11. Stabilization arises automatically; it does not require an extra traditional selection pressure for stabilization. This provides a direct connection between the empirical nature of the evolution of adaptation at the phenotypic level and the nature of genetic change as described by the theory presented here.

12. Evidence shows that rearrangement mutation and point mutation are not random but affected by DNA sequence and structure, and that the determination of mutation is evolving, in accord with the theory proposed here.

13. It is noted that interpreting mutation as ultimately accidental leads to the paradox of the concentration of mutation hotspots in genes that are under pressure for change.

14. Since the theory proposed here holds that mutation is nonrandom and combines information from multiple loci, it predicts that the determination of mutation is complex. Evidence from cryptic variance in the mutation rate across loci and from mutation-recombination hotspots is consistent with this prediction and has no explanation from traditional theory.

15. The above points show that the theory proposed here ties together three grand empirical phenomena that so far have not been connected; these are: sex, nonrandom mutation, and the nature of the evolution of complex adaptation at the phenotypic level.

16. It is proposed that genetic disease can be seen as the result of evolutionary friction points between a long-term process of the evolution of adaptation and a short-term need for performance given the present state of the organism. This addresses the apparent triple association between mutation hotspots, zones of adaptive evolution, and genetic disease.

17. The so-far quintessential example of evolution by random mutation and natural selection—namely the evolution of malaria resistance and sickle cell anemia—is discussed as an example of nonrandom mutation, fitting multiple aspects of the new theory in one (mutations are affected by DNA sequence and structure and show “divergent parallelism”, and the concentration of mutation hotspots relates to adaptive evolution).

18. A more advanced consideration of the new theory shows that alleles play a dual role: alleles participate in the writing of new alleles, and alleles are selected. From this and previous points, several fundamental predictions follow (see below).

19. It is predicted that there must be biochemical activity in the germline responsible for the writing of mutation, and that alleles play different roles in germline and soma—an evolutionary and a performing role, respectively. Activity leading to the *TRIM5–CypA* gene fusion was proposed as an example. These predictions provide a more parsimonious view than that of traditional theory on the fundamental differences between the genetic activity of germline and soma.

20. The existence of mechanisms of mutational writing may inform our understanding of the etiology of cancer.

21. The writing phenotype view holds that there is no dividing line such that on one side of it there are evolved mechanisms that define the range of possible mutations and on the other side of it there is nothing but random accident. The writing phenotype consists of contributions from all levels of taxonomic sharedness and therefore has slow evolving, deeply shared elements that define the phenotype at a general level as well as faster evolving, more specific elements that define the phenotype more specifically, all the way up to “individually determined mutation”—mutation that is determined in a complex fashion with involvement from interacting alleles. In other words, the mutational writing phenotype is simply a phenotype in precisely the same sense that we have been thinking of “phenotype”, except that its role is in mutation writing rather than survival and reproduction. This offers a vastly new way of thinking about evolution. Phenomena like the existence of the genetic code, the fact that mutation is ultimately a creative and not a destructive force in evolution (the mutation rate is not too high and not too low), the existence of the gene duplication machinery, etc., are much better understood from the perspective of the mutational writing phenotype. These phenomena enable evolution as we know it, but from the traditional theory they have been taken as static, exogenous necessities for evolution not themselves understandable by neo-Darwinian evolution. In contrast, here they are considered to be parts of the writing phenotype, and therefore to be as much a part of biological evolution as any other phenomena.

22. The last point leads to the prediction of long-term genetic evolutionary trends not explainable by ns/rm.

23. Selection is not an external judge of phenotypic meaning as in the traditional theory. Instead, it follows from selection on interactions that selection participates in the shaping of the phenotypic meaning of mutation. Thus, mutation is nonrandom both in its mechanism of arising and in terms of its meaning to the phenotype, interestingly bringing together the two ways in which nonrandom mutation is defined.

24. What appears as neutral from traditional theory actually can be subject to selection on interactions and therefore has a vast adaptive potential.

25. *De novo* genes exemplify the paradox of “explaining” adaptive evolution by chance. The writing phenotype offers an indirect route by which selection can exert itself on the evolving *de novo* locus.

26. The questions have been raised of whether there exist sequences with a signature of positive selection but no associated protein (for example in the case of *PIPSL*, and/or in other cases), and whether molecular parallelism could be found in sequences that cannot be subject to traditional natural selection.

27. Epistatic capture amplifies the point above on *de novo* genes and ties it to TEs.

28. TEs may appear as “selfish elements” but nonetheless are a part of an evolutionary system. Novel adaptations may arise from junk DNA but it is not the neutral accumulation of purely accidental mutations that explains them. Biochemical activity in the junk DNA, such as transcriptional promiscuity, may not be simply “noise”. Instead, mechanisms of long-term consequence may appear as an evolved and continually evolving ecology of writing activities active in the germline.

29. The “working sperm” hypothesis has been proposed, according to which much mutational writing activity occurs in the sperm because the large number of sperm balances for the many defects caused by such activity.

30. ENCODE’s results may have indirectly helped to expose the exceptional genetic activity of the germline, part of the nontraditional evolutionary process discussed here.

## Reviewers’ comments

### Reviewer’s report I: Nigel Goldenfeld, University of Illinois at Urbana-Champaign

The main ideas in this long paper are: 

1. Mutation is not purely a random process but contains a deterministic contribution arising from the interaction of genes and from the way in which the organism interacts with its environment.

2. The mutation at a given locus is a function of the alleles at all other loci so is inherently an interacting many-body process. Mutations are not to be thought of as arising from processes acting on individual alleles. This is the sense in which (3) is claimed to be true, thus explicitly avoiding teleology.

3. Selection acts in the canonical way, on combinations of alleles, but these combinations are not disrupted by shuffling due to recombination. Fitness is a collective property of many alleles: a single allele does not have a phenotype and fitness ascribed to it.

4. Sex (here defined in a general way that includes bacterial conjugation, for example) is the rule: obligate asexuality is breakage of sex, the exception not the rule and indicative of a dead end phylogenetically. The problem of the evolution of sex is actually the other way round: sex is the natural result of the evolutionary process as proposed in this paper, and one really needs to ask about the evolution of asex.

5. The writing of mutations from multiple allele interactions into a single locus is itself a phenotype, and interacts with other phenotypes. Consideration of this process corresponds to the notion in classical population genetics of fixation.

6. A number of qualitative predictions are made from the author’s perspective, related in particular to the cost of natural selection, transposable elements, nonrandomness of genome rearrangements and point mutations, *de novo* genes and the origin of life.

If *Biology Direct* is able to publish very long essays, then I think this paper could be published in some form, because its viewpoint is provocative and stimulating.

**Author response:*** I would like to thank this eminent referee for his review. It is exciting to hear that he finds this viewpoint provocative and stimulating.*

I see the above as a good summary of some of the main points of the paper (though naturally, each author prefers his or her own wording), and I have now added my own, more comprehensive point-by-point outline to the summary section of the final version of the paper.

I personally found the article too long, and felt that it could be made more succinct with writing discipline. I am sure many readers will find its title, abstract and introduction too grandiose, so the author should think carefully about that. I hope it will engender discussion and perhaps a vigorous debate with proponents of a more orthodox way of thinking about evolution.

**Author response:*** I also hope it will engender vigorous debate. The previous title and early parts of the paper have been replaced with much better ones, thanks to Professor Goldenfeld’s comments, and the rest has been edited throughout.*

The paper is certainly long; however, I would like it to be able to serve as a reference for future works, as well as show the breadth of evidence supporting the ideas presented here, in order to draw investigators from multiple fields into the discussion.

Many of the examples in the paper are in fields of biology that are beyond my expertise or ability to evaluate critically. I hope that another referee has that level of knowledge.

I think there are some ways to improve this paper. Claims (1–3) above are in some sense consistent with my own prejudices about the evolutionary process, and probably those of some other workers, so I was hoping that the paper would have more quantitative analysis of the issues it raises, to really force a confrontation between theory and experiment, or even new theory versus the status quo. However, that is not the case: this paper is purely descriptive and qualitative. In particular, I would like to know if the author’s perspective makes novel predictions about such phenomena as the rate of evolution, the stress-dependence of hypermutation, or the prevalence of transposable (and other mobile genetic) elements as a function of stress or environmental conditions. These and other phenomena are beginning to be explored quantitatively in the laboratory, so the value of a new perspective will be what it enables us to compute and predict. I would like to suggest that the author try to wrap up this paper with a section that gives a concise and explicit set of predictions, even if only qualitative, and discusses how this perspective could be advanced to the level of a legitimate theory and potentially falsified.

**Author response:*** A quantitative analysis based on the ideas proposed here will indeed be worthwhile, and I believe that it will greatly facilitate the beginning of a new way of thinking about the evolutionary process. While I will be working on mathematizing the ideas proposed here and invite others to do the same, my goal in this paper is different—it is to propose, for the first time, the conceptual foundation that will make such quantitative analysis possible as well as elicit empirical work directly.*

Naturally, those who develop mathematical models tend to focus on the math. But while mathematical modeling is clearly an important tool in biology, the view according to which all that is important is in the math would be too limiting. Darwin’s own theory of natural selection consisted of concepts and empirical observations, showing the power of words. Verbal theory has also been used to great effect by Lorenz, Tinbergen, Doolittle, Brosius and others, and if we do not use it today we stand to lose something.

My theory shows, for the first time, how sex, selection on interactions and nonrandom mutation come together as three aspects of one and the same evolutionary process. Insodoing, it addresses fundamental problems that have been open so far, and raises new predictions and new avenues for research. This theory not only is refutable, it makes strong empirical predictions. Take for example the prediction that sex, defined as the shuffling of hereditary material between individuals by any means, is necessary for the evolution of complex adaptation. One could try to refute it by showing that any one of the putative ancient asexuals has really substantially adaptively evolved or diversified in a purely asexual state. Or take the prediction that there can be no evolution of a complex adaptation ensuring obligate asexuality, but only breakage events leading to obligate asex. One could try to refute it by finding a single true adaptation (as opposed to a breakage event) that ensures obligate asexuality, and opportunities for doing so were discussed. The general-level prediction that mutational writing mechanisms exist in the germ cells can guide further research, indeed in a direction that has not been seen from the perspective of traditional theory, and multiple other questions amenable to empirical investigation have been raised. Quantitative predictions regarding some of the topics that the reviewer mentions may also be drawn from the ideas advanced here, but I believe that they deserve their own, separate treatment.

*In response to the reviewer’s comments above, I have added to the*Summary*section an outline of the main points of this paper, including empirically testable predictions and directions for future research.*

Some specific comments I have are as follows: 

1. Page 4. An important part of the author’s perspective is that information is conserved under allele shuffling. The argument seems to be that information from multiple alleles is combined into one allele, and so is not destroyed by shuffling or even the disappearance of the contributing alleles. How is this different from the well-known concept of epistasis?

**Author response:*** As the reviewer writes, the point of interest is the information-transfer process itself. This paper explains why, because of nonrandom mutation, information is transferred to future generations from combinations of interacting alleles at different loci, despite the fact that the alleles comprising those combinations are continually shuffled. Previous discussions of epistasis do not mention this point, which plays a central role in this paper.*

That being said, the term “epistasis” is very closely related to the phrase “interaction between alleles at different loci” as I mean it in this paper, with three differences that are worth noting. First, traditional theory often conceives of epistasis as a small deviation from a supposed, larger, additive effect. In contrast, this paper does not assume an additive basis for adaptive evolution. Second, traditional theory is concerned with low-order epistasis terms and not high-order ones. In contrast, this paper leaves room for interactions that are highly complex. Third, and critical to the novel point abovementioned, we are used to discussing epistasis in the context of its effect on survival and reproduction only. In contrast, here interactions are discussed not only in such terms but also in terms of their effect on mutation.

2. Page 5. Lamarckism is stated as being impossible but weak forms of it, such as epigenetics, are widely acknowledged to occur. How does DNA methylation for example affect the interactions between alleles in the author’s theory? And what about Landweber’s recent work on the role of RNA in ciliates, which shows evidence for Lamarckian modes of evolution at the molecular level?

**Author response:*** Professor Landweber’s recent work *[256,257]*, like her other work, is fascinating, cutting-edge research. I think that the results from her work, as well as other work on DNA methylation and epigenetics, are in no way contradictory to my paper, and actually would be very interesting to examine from the perspective of the theory proposed here.*

*What I wish to reaffirm prior to proposing a new theory of adaptive evolution is that traditional Lamarckism is not an answer to the question of how multicellular organisms evolve. The reason, as has been articulated well by Haig *[40]*, Koonin and Wolf *[258]* and others, is that we do not expect it to be possible for a mechanism to exist that could sense what the organism needs for improvement at the phenotypic level and then translate it into and implement the needed genetic change that would cause the desired phenotypic improvement in the course of the complex process of development.*

*However, this theoretical block imposed on Lamarckian transmission does not preclude actions at the molecular level from having a heritable effect, and in fact my paper here relies on actions of this sort. The point is that, in my paper, these actions do not “reverse engineer” *[258]* what is needed for improvement at the phenotypic level, but instead are the result of a continually evolving mutational writing phenotype that enables the absorption of information from selection on genetic combinations. Therefore, I find empirical results like those of Landweber and collaborators on actions of heritable effect to be very interesting in the context of the theory proposed in this paper. But I think that the term “Lamarckism” does not apply to my interpretation of them.*

3. Page 7. The section entitled “A prediction following work from Meselson’s lab” needs to be rewritten. Meselson’s work on bdelloid rotifers is described but as a “so-far unofficial result” and indeed references 37 and 38 are to a website and a talk, not to papers. This is strange, because Meselson’s work was published in *Science*, see: Gladyshev et al., *Science* 320, 1210–1213 (2008). Since this result predates the present manuscript, this is at best a “post-diction”, certainly not a prediction.

**Author response:*** Meselson’s interesting 2008 paper in Science, to which the reviewer refers, reports on non-homologous horizontal gene transfer concentrated in telomeric regions *[259]*, whereas, in contrast, here I am referring to Meselson’s recent (and still unpublished at the time of this writing) statements that, after years of having thought the opposite, they found that bdelloid rotifers undergo “homologous gene transfer” *[52]*, taken by Meselson as a proof that bdelloid rotifers are sexual. [*[53]

*Another group has just published in this area *[260]*, and their publication serves to demonstrate an important point about the definition of sexuality. Their evidence suggests lack of conventional meiosis in the individual bdelloid rotifer that they sequenced *[260]*. However, the definition of sex that is of interest for us here is the shuffling of hereditary information at the population level, by any means. Indeed, this group concludes that their results do not exclude “parasexuality”; and that:*

“The high number of horizontally acquired genes, including some seemingly recent ones, suggests that HGTs may also be occurring from rotifer to rotifer. It is plausible that the repeated cycles of desiccation and rehydration experienced by *A. [Adineta] vaga* in its natural habitats have had a major role in shaping its genome: desiccation presumably causes DNA double-strand breaks, and these breaks that allow integration of horizontally transferred genetic material also promote gene conversion when they are repaired. Hence, the homogenizing and diversifying roles of sex may have been replaced in bdelloids by gene conversion and horizontal gene transfer, in an unexpected convergence of evolutionary strategy with prokaryotes.” [260]

Thus, both the statements by Meselson and the statement just quoted call into question the notion that bdelloid rotifers have evolved without sex (without genetic shuffling).

Whether this should be considered a “prediction” or a “retrodiction” at this point may not be the crucial question. Not only are we still far from having a detailed picture of what has occurred in the evolutionary past across the bdelloid rotifers, there is a number of other examples of putatively ancient asexual clades, and in each case my theory predicts that these organisms did not substantially adaptively evolve and diversify without some form of shuffling of hereditary material. This should be testable. Traditional theory, in contrast, does not make a sufficiently serious prediction about the existence of hereditary shuffling as to be refutable by such tests.

4. The “writing phenotype” plays a major role in this article. I did not feel that I came away with a clear understanding of what that is at the molecular level. Given the exquisite knowledge we have now about genome dynamics (e.g. as summarized in ref. [148]) it should be possible to be much more explicit about this, in particular to get to the question posed near the end of the article: how does writing [know how to] process information? (I am deliberately removing the anthropomorphic language here).

**Author response:*** The theoretical and empirical exploration of the mechanisms of the mutational writing phenotype will be exceptionally exciting. But in contrast to the referee, I do not think that analyzing the workings of the writing phenotype is a simple task, despite our current knowledge of genome dynamics. My goal in the current manuscript is to raise the possibility that a new mechanism for evolution exists. Therefore, I am satisfied with positing for the first time that the mutational writing phenotype exists, and with tying it to the problem of sex and the nature of the evolution of complex adaptation, and I leave for future research the vast question of its internal workings.*

5. I would recommend that the title of the paper and indeed the abstract be toned down, and summarise what is actually proposed here rather than the claim to make a new theory. That is, be more specific rather than the rather grandiose but uninformative statement made in the “results” section of the abstract, for example.

**Author response:*** The draft version of the paper that Professor Goldenfeld mentions has been much improved with the help of his comments, though the result is far from perfect. Nonetheless, the reader should know that, with all the qualifications, I am proposing here no more and no less than a new way of thinking about how adaptive evolution works. In that sense, it is a new theory.*

I would like to thank Professor Goldenfeld for his very insightful comments and suggestions for improvement, which I have taken to heart and which I believe have improved the paper substantially.

### Reviewer’s report II: Jürgen Brosius, University of Münster

This is an interesting and thought provoking read containing many “eye-openers” and emphasizing yet unsolved questions concerning the evolutionary significance of sexual reproduction and the proposal of a new theory in harmony with sex.

**Author response:*** It is a great honor to be told by this pioneering thinker that the paper is thought provoking and has many “eye-openers”.*

I would like to thank Professor Brosius for his thoughtful and detailed review. In his comments outlined below, he will attempt to raise difficulties of various kinds with the theory proposed here, including the question of whether the mutational writing process I propose is more like “writing” or “scribbling”; whether there is a “direction” to the mutational process I postulate; how alleles could influence mutation; the role of transposable elements; and more. In the following section, I will answer each question in turn, and explain why traditional theory does not provide a sufficient explanation for the phenomena discussed here.

However, in my opinion, this attempt falls short for a number of reasons outlined below.

Foremost, I would make a distinction between the introduced term “writing” and a possible alternative, namely “scribbling”. Most, if not all aspects the author has covered seem more like “scribbling” rather than “writing” (see below) and despite all the efforts to present presumed examples, I am not convinced of a “writing” process in genomes. It should be noted that this skepticism comes from someone who does not outright reject ‘genome writing’. In contrast, I was among the early voices who considered our recently acquired capabilities to actively write into genomes, including our own in a directed manner as a very significant evolutionary transition: 

“...Homo sapiens, by being able to influence its own genes stands at the brink of a significant transition. We will soon have the ability to use gene therapy to correct genetic disease, clone individuals from somatic cells, introduce desired traits or remove undesirable ones, design genes from scratch and introduce additional chromosomes. Lamarckism is raising its head, after all, albeit without violating the Darwinian principles” (reviewer’s ref. 1).

And: 

“Presently, we are about to witness yet another major evolutionary transition. Through our advances in biology we are now able to transmit knowledge and experimental experience into the germ line of virtually all living species including our own. We will be able to correct the genetic causes of hereditary diseases and implant desired traits into future generations. In 3.5 billion years of evolution, life was perhaps never so close to some form of Lamarckian mechanism as now (...); whether this is a desirable development is, of course, yet another question” (reviewer’s ref. 2).

Prior to the 1970s/80s, all we did was wait for mutations to occur naturally and select for desired traits. There was however an intermediate period last century, when we scribbled by increasing random mutation rates aided by chemicals, UV radiation, X-rays and radioactivity in conjunction with the power of selection in applications such as plant/animal breeding and modification of microorganisms.

**Author response:*** The reader who has started with the reviews before reading the paper should note that the quote above from Professor Brosius’s earlier work, while interesting in its own sake, is on a topic other than the one that is in focus in this paper. Professor Brosius is referring to the process of artificial induction of mutation, whereas the current paper proposes a new theory about how the mutations that occur naturally drive evolution.*

Therefore, the point in his comments above that is of direct relevance to this manuscript is that he is not convinced of a mutational writing process such as I describe here. But it is not a requirement to be convinced at the outset when a new theory takes on such a grand topic as how adaptive evolution works. While I hope that he will eventually be convinced of the mechanism proposed here, the pertinent question for the moment is not whether he is personally convinced, but whether the theory proposed here answers in a parsimonious way questions that have been left open by previous theory and raises new predictions.

Consequently, I can subscribe to the “cranes” concept that includes hypermutability by point mutations or retroposition. Without selective advantage under non-stress situations, lineages that fortuitously kept such mechanisms had a long-term advantage (with hindsight). I have more problems with direction of these processes. Too often, such a “unicorn in the garden” turned out to be a single-horned goat after rigorous experimentation, data analysis, and interpretation (reviewer’s refs. 3–4). Furthermore, during all considerations of directed mutations one has to remember that occurrence of the ‘right’ mutation is one side of the coin—the other is persistence of the mutation. I do not know, but would assume that at least some studies have examined the ratio of neutral mutations nearby (e.g., third amino acid codon positions) versus the ‘right’ mutations.

**Author response:*** My paper does not propose that a mutation is more likely to occur in an environment where it increases fitness than in an environment where it does not, and should not be confused with such proposals. I have clarified this issue in the final draft.*

*Interestingly, the concept of “cranes” gives no sufficient or widely-accepted explanation for evolvability. This concept is defined as a “subprocess or special feature of a design process that can be demonstrated to permit the local speeding up of the basic, slow process of natural selection, and that can be demonstrated to be itself predictable (or retrospectively explicable) product of the basic process” *[113]*. As such, cranes are not well-explained by individual-level selection, and this is well-known. The inventor of this concept addresses this problem in his discussion of sex *[113]* by implying that cranes evolve for a reason that has nothing to do with the evolutionary heavy-lifting that they do—namely, with speeding up evolution. Instead, he implies that they are fortuitously adept at this heavy lifting. But how often can we excuse by fortuitousness fundamental biological phenomena such as the effect of sex on evolution?*

*A more intuitive approach is to argue that cranes arise by high-level selection and that this explains why they are so adept at speeding up evolution. However, it is inconsistent for evolutionary theory to propose, on the one hand, that high-level selection is weak *[23,48]*, and on the other hand invoke it to explain phenomena so important for long-term adaptive evolution as sex, the constructive contribution of TEs, and more. This contradiction in evolutionary theory, where biological phenomena of central interest are either explained by fortuitousness or explained by a theory that is considered weak by many, reveals a fundamental problem unsolved by traditional theory.*

*My proposed solution to this problem (see the section “*Genetic evolutionary trends exist on all timescales*”) warrants attention, because it is distinctly different from both sides of the levels-of-selection debate.*

A key concept described on page 4 and illustrated in Figure 1a of the interaction between alleles at multiple loci being “written” into a further single locus that is being inherited is too vague and hard to understand. Vague, because merely presenting the term “interaction” and statements that alleles from different loci must interact in the determination of mutation fails to give the reader any clue to the molecular genetic mechanisms of allele interactions (alleles merely being variants of genes and not genes or retroposons etc. per se) and how they could modify an additional locus in a heritable mode. It is hard to imagine how one allele combination would “write” (even scribble) differently than another. The reader should be enlightened by examples or at least suggestions of more detailed molecular genetic mechanisms.

**Author response:*** Consider the ethnic effect of malaria resistance mutations, which I discussed in the section “*A quintessential example of ns/rm may be an example of mutational writing*”. One could try to argue that each mutation arises at the same rate in all populations and that different ones are fixed repeatedly in different populations, but that would leave many facts of the situation unexplained. It rather appears that different malaria resistance mutations tend to arise in different human populations, while within a population the same or similar mutations tend to arise repeatedly. My theory is the first to explain this evidence in principle. Now, this evidence suggests that different alleles lead to different mutations. While one is free to say that it is hard to imagine how different alleles (as opposed to genes per se) could lead to different mutations, saying it does not address the empirical data, which show in fact that they do.*

Also, we would not claim that one combination of alleles at different loci could not have a different effect on survival and reproduction than another—that is the concept of epistasis. Why should we think differently of the way that DNA sequence and structure and gene products affect or bias mutation? There is no fact in our entire understanding of molecular and cellular biology that suggests that different genetic combinations could affect survival and reproduction differently but not affect mutation differently.

Regarding detailed molecular mechanisms, it would be very exciting to have them and sooner or later we may have them. However, the goal of the present paper is to set the stage for the exploration of these mechanisms by showing indirectly that they exist. It would take many papers to not only show that they exist but also lay them out in full molecular detail. Furthermore, the lack of molecular detail should not be confused with the lack of a concrete and important high-level outline of the mechanisms, which in fact has been proposed here. Scientists now have the option of going ahead and exploring in detail the mechanisms that have been predicted here at a general level—from the fact that alleles must interact in the biological determination of mutation, to the fact that these interactions occur in the germline, to the possibility that the mutational writing may inform our understanding of the etiology of cancer.

Therefore, I expect the molecular detail to slowly accumulate in the course of future work. Having stated this opinion, I would now like to explain at a higher level why incompleteness of the kind seen here is not a problem for the process of science. While this part of the reply will be lengthy, I believe that it will be informative on the nature of this work as well as the context of biological thought in which it fits.

The very idea of a mutational writing phenotype as articulated here is new. It provides a unifying and parsimonious framework through which to view several heretofore unexplained and important biological phenomena. It shows how sex, selection on interactions, and the nature of mutation come together as different aspects of one process, while raising multiple predictions and directions for future research. At the same time, the mechanisms of mutational writing have not been fully articulated as of yet. This incompleteness may be a block to some readers, but it is also a part of the scientific process.

Traditional evolutionary theory seemed to give us an explanation for evolution that is complete at the level of the essentials. It holds that accidental mutation, random genetic drift, and natural selection together account for evolution. We like to admit that open questions remain, such as the mechanisms of speciation or the role of sex in evolution as seen from a traditional perspective; but those questions are left at some distance from the fundamentals, and in that sense, traditional theory can be said to be complete. But this completeness may be a bit misleading. Which of the available mathematically precise theories truly explains phenomena such as the arising of de novo genes, chimeric genes and the evolution of complex genetic networks organized evolutionarily to a large degree by transposable elements? So far, we have had no explanation for these phenomena but pure chance and fortuitousness as key factors that complete a theory that was supposed to be based on natural selection. But here is the crux of the matter. Saying that pure chance explains the initial arising of de novo genes is a completely well-defined thing to say; it admits no lack of essential knowledge: what occurs by chance no longer needs to be studied, and thus the evolutionary process is presumably completed by chance molecular events. But we must ask ourselves whether this explanation is realistic and satisfying. The vagueness that arises from my theory is due to the fact that, instead of invoking pure chance in addition to an unknown amount of traditional natural selection, I have proposed the beginning of a mechanism, incorporating selection and nonrandom mutation in one unified process. In such a proposal there is much to be asked and to be studied.

Indeed, incompleteness has played a constructive role in the history of evolutionary thought. Darwin’s own theory of natural selection was vague on the mechanisms of inheritance. For this reason it was debated for many decades. It was Fisher and Wright who, borrowing what they did from Darwin, proposed mathematical theories of natural selection. But although their theories have been immensely useful, and though they have given the concept of natural selection a semblance of preciseness, they have left important questions in our understanding of evolution unanswered. In particular, interactions are notoriously difficult to treat mathematically. Furthermore, it is much easier to construct mathematical models under the assumption of random accidental mutation as opposed to nonrandom mutation, because then one does not need to add the structure to the mathematical models that would have been needed in order to describe nonrandom mutation. The current paper holds that selection on interactions and nonrandom mutation are critical for adaptive evolution. Thus, according to this paper, by putting selection on interactions outside of our core understanding of adaptive evolution, and by not examining the possibility of nonrandom mutation, the mathematical models have missed an important part of reality.

*To understand how the history of the field has led us to our contemporary way of thinking, we need to remind ourselves of the problem that Fisher and Wright tried to solve. As alluded to in this paper, Galton *[246]*, Jenkin *[261]* and others never accepted Darwin’s theory of natural selection because of the problem of blending inheritance, which arises in the context of sex. They thought that traits could not persist in the face of sexual reproduction as to be subject to effective selection. In response, Galton proposed the notion of saltation—a big, heritable phenotypic change that on a rare occasion would happen in a single individual and thereafter be untouched by blending *[246,247]*. This avoided use of the ever-present individual variation that Darwin relied on, on account that selection on it could not have a strong heritable effect, and instead relied on rare, big changes—a concept that was later mirrored in Goldschmidt’s “hopeful monsters” *[262]* and that was ultimately rejected. It was Fisher and others who, in the early part of the 20th century, tried to bring back Darwin’s reliance on genetic variation across the population in a manner consistent with Mendel’s laws, or in other words, with inheritance through sexual reproduction; but interestingly, the way this was done shared an important element with Galton’s approach in order to solve the same problem that Galton tried to solve. That is: Fisher’s mutations also do not blend; they also bring about phenotypic changes that are supposed to occur in single individuals by chance one day and continue undisturbed from then on despite the sexual shuffling of genes. Like Gaton’s saltations, they are independent changes, but “small” ones that can sum up. This smallness was supposed to make them more likely to happen by accident, though at the heart of the matter, we still have no quantification of the supposed Fisherian “smallness” of chance, not to mention that we are no longer using it in an internally-consistent fashion (see introduction to this paper and later discussion in this review). As discussed in this paper, Fisher’s mathematical framework of additive effects did not involve interactions as the drivers of evolution, and it did not actually explain the sexual shuffling of genes, but rather neutralized it; that is, from the Fisherian view, the shuffling of the hereditary material is not necessary.*

In this paper, we have gone back to these deep roots of the modern synthesis—to the basic assumptions on which the modern theoretical approach stands. I have proposed that the shuffling of the hereditary material is of necessity for the evolution of complex adaptation; that it creates a wide variety of combinations of interacting alleles at different loci that are then selected; and that a complex adaptation arises by a process of “convergence” as described in the paper, where information from combinations of alleles at different loci is put together by the writing of mutations. In other words, inheritance involves more biological detail than we had thought: it involves the heredity of mutations that combine information from transient genetic combinations. This amounts to a new connection between selection on the phenotype and genetic evolutionary change.

We can now see both how the theory proposed here does better than Fisher and Wright’s theories in some ways and why it is incomplete at the same time. The additive-effects–based connection between selection on the phenotype and genetic evolutionary change that Fisher invented, while being perfectly crisp and immediately amenable to mathematization, did away with individuals as complex wholes. It encouraged instead the very crisp perspective that adaptive evolution is based on supposedly accidental mutations that are normally beneficial as single units. In contrast, the present theory offers a connection between selection on the phenotype and genetic evolutionary change that allows for the first time selection on interactions to have a direct hereditary effect and thus drive evolution. It allows selection on complex wholes. This connection is not only more consistent with the long standing intuition of biologists that interactions must be critical for the evolution of complex adaptations, but also resolves some of the current mysteries brought about by the genomics revolution (see paper). Now, this development leaves open the question of the detailed nature of the mutational writing mechanisms, and thus makes the theory vague on an important point. But while the theory as a whole is currently more vague than Fisher’s theory, importantly, it is less vague than Darwin’s (since Darwin’s theory had no mechanism of inheritance), and it solves the problem at the basis of the modern evolutionary synthesis in a deep way that is more in line with Darwin’s own observations than the way of Fisher and Wright (see, for example, the connection between “divergent parallelism” discussed here and Chapter V of the Origin of Species).

*In fact, Darwin’s theory was vague not only on the mechanism of inheritance. It was also vague on the central question of the causes of variation. In the beginning of Chapter V of the Origin of Species Darwin wrote: “I have hitherto sometimes spoken as if the variations... were due to chance. This, of course, is a wholly incorrect expression, but it serves to acknowledge plainly our ignorance of the cause of each particular variation” *[60]*. Until now, this incompleteness has left a big hole in our thinking about evolution.*

Interestingly, in this paper, Darwin’s two great vague and incomplete areas—that of the mechanism of inheritance, and that of the causes of variation—(and in fact other mysteries left open by him, such as the question of why sex exists) are put together into one: nonrandom mutation is part of the nature of inheritance and allows selection on complex wholes; the cause of variance and the nature of inheritance are not separate things. But in putting them together, this work opens a vast new vague area—the mechanisms of mutational writing.

It is surprising that the author did not even mention the term “epigenetic(s)” once in the entire manuscript (except in about 3 references).

**Author response:*** To examine epigenetics from the perspective of the present theory would be of great interest, and I agree that the relevance is clear. Having had already tended to so many topics, I had reluctantly decided to leave this one for future research in the writing of the first draft. I have now added a very brief mention of it in the summary, thanks to Professor Brosius’s comment. This brevity stands in no relation to the importance of the topic and should not be seen as disinterest or belittling of it.*

Perhaps an important explanation for the role of sex in evolution might be the fact that TEs would not be successful without sex (reviewer’s ref. 5). About a decade later, after the occasional beneficial effects of TEs on genomes began to emerge, the same author wrote: “It has been shown that molecular symbionts (such as transposons and plasmids) derive a major selective advantage from conjugation and sexual outbreeding” (reviewer’s ref. 6); see also (reviewer’s ref. 7). This is impressively documented by the rapid spread of P-elements in the *Drosophila* genus (reviewer’s refs. 8–10). While in the short run, asexual species might have a selective advantage, in the long run only those lineages survived that happened to maintain sexual reproduction—sex being a relict from as early as the RNA world [54,55]. As an aside, bdelloid rotifers might have been sexless for over 35 million years, but instead, they have been involved in rampant horizontal gene transfer, which, if not sex, is an efficient substitute [259]. The interesting concept of Michael Ghiselin that a species should be considered an individual should also be discussed in the context of the evolutionary significance of sex (reviewer’s refs. 11 and 12).

**Author response:*** I disagree with the traditional approach according to which sex can be explained by some particular secondary benefit or a conglomeration of those. In the above and in the below parts of this review, the reviewer suggests that an important explanation for the role of sex in evolution could be that TEs are only successful with it; that the reason why many plants are at the outcrossing end of the spectrum but few are at the selfing end might be due to better geographical dispersal of alleles or allele combinations (see below); and that de novo genes fortuitously arise from the transcriptional noise generated by transcriptional promiscuity (see below).*

As legitimate as these hypotheses may be, the strength of my theory is in providing a unifying approach: it explains the role of sex in evolution, how selection on interactions can drive evolution, why the evolution of complex adaptation appears as it does at the phenotypic level, why there is divergent parallelism at both the molecular level and phenotypic levels, why transcriptional promiscuity exists in the first place, why there are few species at the outcrossing end, and many other things. A unifying perspective engages the data better than a perspective that treats problems in isolation from each other, and it opens up new directions for research that cannot be seen from the latter.

Other points:

Using the term “convergence” in its dictionary meaning of “moving toward union or uniformity” might lead to confusion. A few pages down, the reader might slip back to the established meaning in evolutionary biology. Perhaps a term “comulgation” from the Spanish language “comulgar”, meaning “to share, to communicate” could be introduced. Unfortunately, it also has a religious use: http://www.wordreference.com/es/en/translation.asp?spen=comulgarhttp://dictionary.reverso.net/spanish-english/comulgar From my own experience, though, I have to point out that it is difficult to introduce novel terms, however useful.

**Author response:*** Following much consideration, I have decided to leave the terminology as it is, while fully recognizing the importance of Professor Brosius’s point.*

Concerning the statement: “...the farther we get in time from the early generation, the more the basis of information in the early generation comes to be shared by individuals” the author should consider that new alleles are constantly being formed as well.

**Author response:*** Agreed, and this has already been taken into consideration—see Figure *2.

The reason why in plants many species are at the pure outcrossing end, and yet very few at the pure selfing end might simply be due to better geographical dispersal of alleles or allele combinations (see above).

**Author response:*** See response above to the comment on the role of sex in evolution and TEs.*

The locus of retroposition is, apart from a preference for ubiquitous A/T-rich target sequences only determined by complementarity of the retroposed RNA 3’-end and a ragged-ended DNA strand for priming. Since there are a number of tailless SINE elements, priming could, in theory, occur at any sequence in the genome (reviewer’s ref. 13).

The introduction to the first sentence of the second paragraph, page 24 is not quite correct. Of course, there is rearrangement between TEs and the cut-and-paste mechanism of DNA transposons is some sort of rearrangement. However, retroposition is more like a duplication of—if not genes—but of genetic elements and it is quite random.

**Author response:*** I consider “duplication” to be included under the notion of “rearrangement”. It is easy to call “quite random” things we do not understand, and which could be the result of neither pure accident nor an omniscient process, but rather the result of a decentralized writing system—an ecology of writing activities.*

Also, it should be kept in mind that the “donation of every kind of functional element” mostly requires additional and fortuitous mutational steps that may take tens of millions of years to occur and if they occur, they might not persist, because they still might be neutral or only slightly advantageous (reviewer’s ref. 14).

Concerning *de novo* gene evolution (pp. 30/31), the author states: “First, a previously complete and active gene is duplicated by a single ‘duplication mutation’ all at once along with its regulatory and coding sequences”. This is only partially true: retroposition matches existing mRNA reverse transcripts with novel regulatory elements (reviewer’s ref. 15).

**Author response:*** The point that I am making is not that only the whole gene duplication route is available to new genes, but that only this route is consistent with the traditional idea of the gradual accumulation of small-effect chance mutations under traditional natural selection, and that it may be imprudent to dismiss the other routes, which are inconsistent with this traditional idea, as the result of pure chance without much thought.*

Concerning *de novo* genes additional references should be cited [199,201].

**Author response:*** References added.*

Much more common is the exaptation of novel gene parts from retroposons or actually any neutral sequence (reviewer’s refs. 16–20).

**Author response:*** I agree with the importance of movements of genomic pieces and ask whether mechanisms (indeed, evolvability) or only pure chance are involved in their movements.*

Re-wiring of the gene regulatory landscape of endometrial stromal cells (ESCs) of the placenta, if true, only can be a random process. If 1,500 MER20 elements were recruited into this regulatory network, what about the remainder of the 15,000 MER20 elements in the human genome? I highly recommend the critical reader to look at the chapter (actually the entire manuscript is excellent) entitled “Transcription factor binding does not equal function” by Dan Graur and colleagues [7]. Furthermore, although Lynch et al. [15] could show with reporter constructs ex vivo that MER20 elements respond to progesterone/cAMP in ESCs, it is only part of the confirmation of a regulatory network. The problem with these and similar studies is, that current science politics might grant us the time to prove a working hypothesis but not to falsify it (reviewer’s ref. 21). Not many laboratories can afford the leisure to test the influence of TEs on gene expression by costly and time-consuming targeted deletions in mouse or other animal models.

**Author response:*** What we are concerned with here *[15,205,206]* is the evolutionary organizing by transposable elements of more than 1500 genes into a new genetic network underlying a novel, complex adaptation that is the decidualization of the endometrium. It is not that we had not known before that TEs play a constructive role in evolution; it is rather the massiveness of this example that is intriguing. This work comes out of Günter Wagner’s lab, who has been a leader in evolutionary biology, pushing the envelope on our understanding of evolution throughout his career. These results provide strong p-values for the nonrandom association of MER20s with this network, and in my opinion they are quite challenging as they are.*

The problem that these results raise is as follows. If one were to explain from traditional theory the evolution of a network of this sort, the main way of doing so would be to say that it is due to some mix of selection and neutral evolution. But how much fortuitous chance would be involved in such a mix? How many neutral movements of TEs and neutral mutations in them had to take place before something was established that could be subject to traditional natural selection and explain the arising of a new network, if we operate under the assumption that it is accidental mutation and natural selection that explain things, and does this explanation make sense?

The reviewer argues that, assuming that 10% of MER20s are involved in tying together this network, and that the rest fall elsewhere, it must have been a random process that gave rise to this network. Perhaps this argument would have been true if the only alternative to accidental mutation were an omniscient process that frugally used each type of TE for one purpose; but according to this paper, this is not the only alternative.

In fact, saying that the arising of this network must have been a random process is problematic. At its core, the traditional view of adaptive evolution holds that small chance-events occur, and selection pulls out of the noise beneficial changes, which can thus accumulate and create an adaptation. When faced with evidence not fitting with this view, such as de novo genes, this view forces us to argue that it is still just a small genetic change that arises by a sequence of fortuitous chance events absent selection (a small whole gene, in this example), and that it could happen by accident after all (we will see if this approach is valid below). But in the example discussed here we have the evolution of a network of more than 1500 genes that come together to underlie a complex, novel adaptation. Therefore, the question that this example helps us highlight is this: Where do we draw the line? When is the amount of accidental chance that we invoke for explaining the evolution of complex adaptation from the traditional view too much, and when is it not too much? The answer that we are currently using is obvious: the line is defined post hoc so that it always includes every empirically discovered case of evolution as one that could in principle be explained by traditional accidental mutation, random genetic drift and some unquantified amount of selection. These post hoc explanations harbor the double standard of saying that we have an explanation for the evolution of adaptation, in the form of a process where natural selection inexorably tests many mutations, among them beneficial ones, each of a slight enough benefit that could presumably have arisen by accidental chance, while at the same time invoking just as much additional chance as the situation requires, in the form of neutral evolution that also happens to play an inherent role in the evolving adaptation (this invocation of additional chance undoes the whole point of the frugality of the reliance on chance, a frugality which was supposed to be the anchor of the scientific explanation). This double standard is a severe problem with the traditional view of adaptive evolution.

*We are therefore led to ask whether there are mechanisms involved in the present example beyond those considered by traditional theory. I would agree with the view that the proliferation of TEs of the same kind already equipped with cryptic binding sites *[205,206]* could subject many genes to activation by the same transcription factors and is therefore a very good way of tying those genes together *[15,208]*. But note that this view makes TEs inherently useful for evolution in a mechanistic way, and this prepotency is more congruent with the theory that I have proposed here than with traditional theory.*

Two to three decades ago, it was extremely difficult to convince the scientific community about occasional exaptations of a TE or part thereof into a novel function. More recently, we have the opposite problem. Namely, that there are many attempts to sweepingly assign functions to the majority of TEs in the genome. While it is clear that we still have a lot to learn about the grammar of genomes, trying to read too much into their structures is somehow reminiscent of tea-leaf reading akin to the “bible code” (reviewer’s ref. 22).

**Author response:*** My paper does not suggest that all or most TEs play a role in survival and reproduction. What I have suggested is in line with Fedoroff’s view that TEs are inherently useful for evolution *[19]*. It does not follow that the majority of TEs have functions in the performing phenotype at any given point in time. The usefulness of TEs for current survival and reproduction and their usefulness for evolution are two different things *[211]*.*

A similar problem concerns the detection of RNA transcripts from >60*%* of the human genome by ultra-deep RNA sequencing which leads to absurdities of equating function to any aberrant RNA snippet and hence claiming, as ENCODE recently did, that most “junk” DNA is functional indeed. The author appears to fall into a similar trap: “Transcriptional promiscuity is a highly involved mechanism, requiring the orchestration of a complex machinery to both create and compensate for the pattern of expression [212], and its evolutionary origin is a mystery”. Others would call it basal levels of transcription or insufficient transcriptional silencing or read-through transcription or spurious transcription initiation and elongation etc. ([7] and reviewer’s ref. 23). It simply is an imperfection akin to point mutation where replication is not completely error-free. However, I agree with the notion that such transcripts have the potential to fortuitously lead to novel genes encoding functional RNAs, or even protein coding mRNAs out of these spurious mostly low-level transcripts (reviewer’s ref. 23. and [192]).

**Author response:*** The reviewer touches here on an important difference between his view and mine. His view is that TP is error, and that mutation is error. My view is that TP is hard to explain biologically as an error, because it requires evolved adaptations to compensate for it *[212]*. I hold that this fact, along with its ability to allow interactions in the writing of mutations as predicted by my theory, makes TP an intriguing phenomenon.*

*From the perspective of the theory proposed here, many different observations fall into place as pieces of one puzzle, including transcriptional promiscuity, molecular parallelism, the nonrandomness of mutation that comes out very clearly from the empirical evidence, and much more, as already stated in the above comments and in the paper. From the traditional theory, these things are dismissed one by one: mutation is a random accident (despite all the evidence to the contrary—traditional theory does not provide an answer to the paradoxes that I have elaborated on in this paper); mutation hotspots just fortuitously happen to be in loci undergoing concentrated adaptive evolution; transcriptional promiscuity is just an error that happens to take place, of all places, in the cells where it can affect evolution, and, fortuitously, it is not disruptive; TEs fortuitously acquire functions, so much so that a notable percentage of TEs of a particular kind have played a substantial role in the evolutionary organizing of a complex network of more than 1500 genes; the incredible evolutionary usefulness of TEs is then explained *[8,119]* as being partly the result of extremely high-level selection, even while the effectiveness of high level selection is far from being widely accepted, for basic reasons *[23,48]*; extensive molecular parallelism just happens to happen, and it is then simply assumed that the mutation rate is high enough to allow it to happen, even while cases of parallelism such as the independent arising of the adaptive TRIM5–CypA gene fusion in different monkey lineages *[105,107-112]* show that the assumption of random mutation is faltering *[105]* (not to mention the curious connection of the high expression level of the CypA gene in the germline *[116]*, which is precisely in accord with the theory presented here), and indeed no calculation is provided by traditional theory showing that accidental mutation can account for such cases; mutational mechanisms are reported and labeled as “deeply perplexing” *[155]*, are discussed here at length in connection with organismal-level observation crucial to Darwin, but again these observations have been put aside by traditional theory for the lack of a suitable theoretical framework; and so on and so forth. This approach dismisses many critical observations and explains away others in an ad hoc fashion, whereas, in contrast, the present paper provides a unifying framework that seriously engages the findings and that opens up new avenues for future research.*

Comment concerning the statement “To address *de novo* genes from a traditional viewpoint, it is said that Jacob did not know that there is so much transcriptional ‘noise’...”

Above all, Jacob did not know that genomes of multicellular organisms contain so much non-functional DNA as cradle or cauldron for *de novo* genes.

and

“This example shows that there is a severe problem of lack of quantification of the amount of random chance that we call upon, not to mention such facts as that the *Poldi de novo* gene arose with an existing alternative splicing pattern [11].”

This is no surprise, as a novel gene is not expected to arise with perfect splice sites; hence alternative splicing patterns are common.

**Author response:*** I argue that there is no calculation that shows that it is reasonable to expect genes to arise de novo, even accounting for the large size of the pool of transcripts to draw from, and the quotations above are taken from the part of my paper that makes this argument.*

*Some would dismiss de novo genes as no surprise, on account that de novo genes are small enough, and that the pool of transcripts to draw from is large enough, so that it is reasonable to assume that once in a while one of the transcripts will find use as a new gene by chance. But the Poldi gene is 853 nucleotides long with exon 2, and 785 without. There are 4*^785^ random genetic sequences of this smaller length. This number dwarfs the number of atoms in the visible universe, and thus also dwarfs the number of transcripts available due to TP. The question therefore is not whether de novo genes are “sufficiently small” or whether TP provides “so many transcripts”—these statements do not address the challenge that the evidence has brought forth. The question is what fraction of random sequences of such sizes will be useful in any given organism. The literature does not provide an answer to this question. There is no hint of a calculation or empirical evidence showing that a de novo gene can arise fortuitously without involvement of selection. Our lack of ability to answer this question from traditional theory should be acknowledged as a problem. In contrast, my theory begins to address this issue, by saying that there are mechanisms in place that enable the evolutionary route taken by de novo genes, mediating between them and selection. Interestingly, my theory argues that TP is one of these mechanisms.

In trying to explain de novo genes in a way other than just by saying that they arise by pure chance, one might argue that there must be smaller intermediates on the route to a de novo gene, and that those intermediates were somehow subject to natural selection. I would agree with this line of reasoning, but add that if one wanted to explain the arising of an intermediate in a de novo manner, the same question would apply again. The number of random genetic sequences only 50 nucleotides long still dwarfs the number of transcripts available due to TP. Furthermore, de novo pieces are a problem all the way down, since at some point the many de novo pieces also need to be connected together, and that would require again an unquantifiable amount of pure chance according to the traditional view.

I would like to thank the reviewer again for sharing his highly informative views and his expert knowledge, which greatly helped to explore points of difficulty and made a very important contribution to this work, as well as for other helpful comments of his not included in the above, all of which I have taken into consideration.

### Reviewer’s report II: reference list

1. Brosius J: **From Eden to a hell of uniformity? Directed evolution in humans. ***Bioessays* 2003, **25**(8):815–821.

2. Brosius J: **Disparity, adaptation, exaptation, bookkeeping, and contingency at the genome level.*** Paleobiology* 2005, **31**(2):S1–S16.

3. Stahl FW: **Unicorns revisited.*** Genetics* 1992, **132**(4):865–867.

4. Smith KC: **Spontaneous mutagenesis: experimental, genetic and other factors.*** Mutat Res* 1992, **277**(2):139–162.

5. Hickey DA: **Selfish DNA: a sexually-transmitted nuclear parasite.*** Genetics* 1982, **101**(3–4):519–531.

6. Hickey D: **Molecular symbionts and the evolution of sex.*** J Hered* 1993, **84**(5):410–414.

7. Wright S, Finnegan D: **Genome evolution: sex and the transposable element.*** Curr Biol* 2001, **11**(8):R296–R299.

8. Good AG, Meister GA, Brock HW, Grigliatti T, Hickey D: **Rapid spread of transposable *P* elements in experimental populations of* Drosophila melanogaster*.*** Genetics* 1989, **122**(2):387–396.

9. Engels WR: **The origin of *P* elements in* Drosophila melanogaster*.*** BioEssays* 1992, **14**(10):681–686.

10. Silva JC, Kidwell MG: **Evolution of* P* elements in natural populations of* Drosophila willistoni* and* D. sturtevanti*.*** Genetics* 2004, **168**(3):1323–1335.

11. Ghiselin MT: **A radical solution to the species problem.*** Syst Zool* 1974, **23**(4):536–544.

12. Ghiselin MT: *The Economy of Nature and the Evolution of Sex.* New York: University of California Press; 1974.

13. Schmitz J, Churakov G, Zischler H, Brosius J: **A novel class of mammalian-specific tailless retropseudogenes.*** Genome Res* 2004, **14**(10A):1911–1915.

14. Krull M, Petrusma M, Makalowski W, Brosius J, Schmitz J: **Functional persistence of exonized mammalian-wide interspersed repeat elements (MIRs).*** Genome Res* 2007, **17**(8):1139–1145.

15. Brosius J: **Retroposons–seeds of evolution.*** Science* 1991, **251**(4995):753.

16. Lev-Maor G, Sorek R, Shomron N, Ast G: **The birth of an alternatively spliced exon: 3’ splice-site selection in* Alu* exons.*** Science* 2003, **300**(5623):1288–1291.

17. Singer SS, Männel DN, Hehlgans T, Brosius J, Schmitz J: **From “junk” to gene:* Curriculum vitae* of a primate receptor isoform gene.*** J Mol Biol* 2004, **341**(4):883–886.

18. Krull M, Brosius J, Schmitz J: **Alu-SINE exonization: en route to protein-coding function.*** Mol Biol Evol* 2005, **22**(8):1702–1711.

19. Möller-Krull M, Zemann A, Roos C, Brosius J, Schmitz J: **Beyond DNA: RNA editing and steps toward* Alu* exonization in primates.*** J Mol Biol* 2008, **382**(3):601–609.

20. Baertsch R, Diekhans M, Kent WJ, Haussler D, Brosius J: **Retrocopy contributions to the evolution of the human genome.*** BMC genomics* 2008, **9:**466.

21. Popper K: *Unended Quest: An Intellectual Autobiography, Karl Popper.* London: Routledge; 1993.

22. Drosnin M: *The Bible Code.* New York: Simon & Schuster; 1997.

23. Brosius J: **Waste not, want not–transcript excess in multicellular eukaryotes.*** Trends Genet* 2005, **21**(5):287–288.

### Reviewer’s report III: W. Ford Doolittle, Dalhousie University

I confess that I found this a very irritating essay, and several times nearly gave up reading it. It is clear that Prof. Livnat has thought and read much and deeply about evolution and does seem to be offering hope for a new conceptual framework within which to rationalize observations that many claim to find puzzling. He admirably summarizes a vast number of phenomena which neoDarwinists have to stretch themselves to rationalize, and argues that these are “consistent with the present [that is, his] theory.”

But I’ll be damned if I can tell you just what exactly that theory is. Livnat packages a variety of accepted observations about epistatic interactions as these affect not only gene expression but also mutation under the notion of “writing”, but these seem to me not much different than the sorts of things people are referring to when they write about “evolvability” and claim (reasonably enough) that it too evolves. Nobody thinks that mutations are not complexly caused, nor that evolution does not impinge upon mutational mechanisms and their specificity. Nor does any sensible contemporary neoDarwinian deny that the complexity with which function is determined in a genome such as ours has important effects on the direction and speed with which it evolves.

Most interesting, and possibly the core of the “present theory”, is the notion that although synergistic interactions between multiple alleles at unlinked loci brought together by sex and recombination are transient (because of sex and recombination), they may by their joint mutational effect on some third locus result in a novel and potentially useful new allele which in its singleness can be a permanent contribution (not broken up by recombination). So sex is important, indeed foundational for evolution, because it creates advantageous new genes that are immune to it. Maybe this is an interesting new way of looking at things: time will tell.

**Author response:*** Addressing the problem of sex is no small matter. The reviewer acknowledges the novelty of the hypothesis that lies at the core of the theory that I have proposed here and writes that it would make sex “important, indeed foundational for evolution,” and that maybe it is “an interesting new way of looking at things”.*

The reader familiar with previous hypotheses on the role of sex in evolution should note that this hypothesis is very different from the previous ones, because it connects sex and mutation while implying that the nonrandom aspect of mutation is critical: it allows selection to act on combinations of alleles at different loci as interacting wholes while having a heritable effect.

We now need to pursue the consequences of this hypothesis to see how they address the reviewer’s questions.

Traditional theory only defines random mutation with respect to its effects on immediate fitness. As evolutionary biologists, we are quick to admit that, in other senses, mutation need not be random, and that various complicating factors may cause the mutation rates to be higher in some places rather than others, or even affect what mutational change will take place. But the question that my paper raises is whether these complicating factors are or are not of profound consequence for our understanding of the process of adaptive evolution. My paper holds: “yes, they are”.

*The reviewer writes that no one would dispute that evolution impinges upon mutational mechanisms, and indeed some of the most inspiring papers in population genetics have been written on the evolution of modifiers affecting the mutation rate or the recombination rate *[61-63,263-268]*. But the ways in which evolution is thought to impinge upon mutational mechanisms are not a systematic part of our traditional explanation of how adaptive evolution happens at its core. The core is that of ns/rm plus drift, and on top of this basis various effects have been modeled. This traditional core is very different from what I am proposing here. To make this explicitly clear, the traditional idea of the mutation that drives evolution is that mutation is the result of accident. From that perspective, the complicating factors that Professor Doolittle claims no one disputes are, to our deep understanding of evolution, complicating factors. They are not front and center. In contrast, my theory states that the mutation that is of relevance for the evolution of complex adaptation is not the result of accident, but the outcome of an evolved and continually evolving biological phenotype. What were previously considered “complicating factors” are actually the basis of things. The possibilities for ongoing mutation are defined through genetic interactions, and this fact is at the heart of the evolutionary process.*

Thus, while the reviewer writes that nobody would dispute that mutation is “complexly caused,” the question at hand is whether these complex genetic influences on mutation are a fundamental part of the adaptive evolutionary process. If sex becomes foundational according to the possibility that the reviewer recognizes, then at the same time these epistatic effects on mutation become foundational, because they are the ones that underlie this hypothesis on sex in the first place.

Note that, according to this view, there is no selection acting on accidental variation that, by acting indirectly on modifiers, or by favoring some higher level entities (such as species or clades) over others, evolves evolvability, because adaptive evolution is not based on traditional accidental variation in the first place.

*The key to understanding this paper with regards to evolvability is understanding the full implication of the assumption that mutational writing can be seen as a phenotype, and that there is no adaptive evolution but the joint evolution of the writing and performing phenotypes. As explained in the section “*A more detailed look into the new theory*”, if mutational writing is a phenotype, then this immediately implies that it would include both taxonomically shared phenomena that define the possibilities for genetic change at a general level, such as sex and recombination, as well as more specific influences on the possibilities for change, up to and including the individually varying epistatic influences on mutation that figure into the hypothesis on sex that the reviewer recognizes. This means that we do not treat evolvability as a secondary issue: inference of the writing phenotype from the many pieces of evidence discussed in this paper implies evolvability directly. Importantly, there is no longer a question from this perspective of how the writing phenotype (and thus evolvability) evolved independently of other biological structure, as though we are still looking for an explanation of origins in an ns/rm core. The mutation that drives evolution has always been the outcome of biological actions, and this biological activity from the “beginning” has evolved along with the performing phenotype to its present state.*

This of course ties to the view that sex is original.

Given this theory, our understanding of evolvability is improved. When trying to explain, from the traditional theory, the evolvability provided by such phenomena as sex, recombination and an evolutionarily productive rate of mutation, there is a problem. Evolvability, by definition, is something that facilitates population-level evolution. It is not a property of an individual, because an individual does not evolve; populations do. The individual does not benefit in terms of its own fitness as compared to the fitness of other individuals in the population based on how evolvable the population that it belongs to is. Therefore, how can traditional natural selection acting on individuals lead to the evolution of evolvability?

*Working from the traditional theory, one possibility is to propose that evolvability evolves not based on individual-level selection, but based on selection at the level of groups, species, or clades (e.g., *[269]*). However, high-level selection is considered by many theoreticians to be weak *[48,263]*. Therefore, to be forced to explain complex biological phenomena that are of much importance for evolvability by applying high-level selection is to be in a weak position. I will discuss this in detail later.*

*Another possibility is to address evolvability through modifier theory *[32]*. While this theory avoids high-level selection entirely, it also recognizes the problem that evolvability is not necessarily favored in the process of individual-level selection and is a priori agnostic on what outcome to expect *[32]*. In this approach it is assumed, for example, that one locus controls the mutation rate at another locus or the recombination rate between two other loci, and a model is constructed to examine the evolution of allele frequencies in that gene *[61,62,263]*. Note that these models do not require epistasis in the determination of the modifiers’ action (whereas my theory requires it for the core hypothesis on sex, highlighting a difference in mechanism). More importantly, these models presumably have been interpreted as though the mutational cause of the alleles in the modifier locus and in the other loci is accidental.*

*This important modeling framework has actually exposed a difficulty in evolving evolvability within the traditional framework, namely the reduction principle *[61-63]*. This principle shows that traditional natural selection indirectly affecting modifier loci is actually often expected to decrease mutation and recombination rates (to shut down evolvability) *[32,61-63,263-266]*. Conditions can be found where the opposite happens, and the behavior of the system is complex (e.g., *[267,268]*). But ultimately, this modeling framework does not provide a systematic solution to the evolution and maintenance of evolvability based on ns/rm *[32,266]*. Quite the contrary, I would argue that its greatest contribution was in showing the lack of such a solution from the traditional theory *[32]*, thus highlighting the problem of evolvability for traditional theory.*

According to my theory, we do not need to look for circuitous explanations for evolvability from an ns/rm core. Inference of the writing phenotype from the many empirical observations discussed in this paper immediately implies evolvability, and the writing phenotype did not evolve from ns/rm, but rather, just like the performing phenotype, has been around as long as life has been around, and both have become more elaborate. This explains evolvability in the sense that it now becomes yet one more of many pieces that are connected in a parsimonious framework.

Why I didn’t give up reading was because Livnat considered but then dismissed the sort of levels-of-selection explanation for the evolvability of evolvability that I myself endorse (his references [8,16] and [119]), thus giving me the opportunity to put in a word for it in this review. He writes that “TEs [transposable elements] can have the appearance of selfish elements yet be an inherent part of the mutational mechanisms that serve the evolution of organisms”. I would say that transposable elements are selfish and spread at the genomic level because of that, though they are disadvantageous at the level of individual organisms within species, where they are selected against. But because possession of these mutational agents may speed speciation or delay extinction at a still higher level (species?), they can be seen as advantageous and selected for at such a level.

Livnat equivocates on this view, indeed rather cops out. He writes that “Doolittle offered a way of resolving this conflict, by proposing that clade-level selection is responsible for the existence of a system hospitable to TEs due to their long-term usefulness. But the debate over whether selection at levels above the gene and individual is strong enough to affect such things is far from resolved. Hence we admit that the question is open”. Most biologists know that group selection as espoused by Wynne-Edwards is in very bad odor and shy away from invoking it. But surely if species or higher taxa differ in their evolvability because of TE accumulation they will be differently successful in the long run even if the TEs spread because of selfishness. Maybe more theory needs to be developed here, divorced from issues of altruism and complex population behaviors (the usual battleground). So it is true that the “question is open”, but that phrase should not be read as dismissive.

**Author response:*** I do not mean to equivocate on this issue, and I will make further clarifications here. In his writings *[16,119]*, Professor Doolittle acknowledges the problem of high level selection. As Williams *[48]* argued, selection at levels higher than the individual is much weaker than selection at the level of individuals for the following reason. There are many generations of individuals, and many individuals in each generation. Selection has much to choose from, and is therefore strong. By comparison, how many groups are there, and how often do they give rise to “offspring groups?” Likewise, the turnover rate of species is incredibly small relative to the turnover rate of individuals. Selection has far fewer opportunities to act, and thus the potential for the buildup of an adaptation by high-level selection is far smaller than that which we expect from individual-level selection. Make no mistake: by reminding the reader of Williams’s argument, I do not mean to imply that I am a supporter of Williams’s world-view, which I am not. Rather, what I mean to state is that, as long as we work from the framework of traditional theory, that argument is still relevant, and assigning importance to the explanatory value of high-level selection proposals is not a straightforward matter.*

*Now, I do not disagree with Professor Doolittle that TEs can be described as though they are selfish elements on the molecular level, with costs at the individual level and long-term usefulness for evolution. However, I am concerned with the question of what explains the fact that they are useful in the first place, evolutionarily. Using only the past arsenal of ideas, one possibility is to propose that the system of TEs and their regulation are fortuitously useful in the long-term, and that once they are there, high-level selection plays some role in their prevalence *[8,16,119]*. Another possibility is to suggest that high-level selection has gradually built up the system of TEs and their regulation and thus explains the origin of their long-term usefulness. One could also combine these possibilities, proposing an initially fortuitous usefulness and further gradual elaboration of it by high-level selection. Doolittle’s arguments in *[16,119]*, emphasized in the context of the evolutionary usefulness of introns through exon shuffling, but also discussed as applicable to TEs, raised the first possibility, and a parenthetical note in his 2013 paper *[8]* (page 5298) may also be read as consistent with the third. While considering these options, Professor Doolittle consistently admits the relative weakness of high-level selection, implying the untenability of maintaining a trait by high-level selection in the face of noticeable individual-level costs, and he carefully constructs his arguments in a manner that recognizes this problem. In the case of TEs, he relies on TEs being selfish elements at the gene level to provide their own proliferation and maintenance, thus countering from below the individual-level selection pressure against them (in other words, TEs are favored at the gene level (strongly), selected against at the individual level (strongly), and selected for again at the species or clade levels (weakly)). However, as more empirical results are obtained, the more we see that the contribution of TEs is immense, and that their regulatory system is complex *[15,19]*. To me, these findings make it increasingly unappealing to explain the origin of the usefulness of TEs by fortuitousness. They also make the question more pressing of whether high-level selection can gradually build up a complex system with notable long-term benefits despite its relative weakness. In the explanations above, only fortuitousness and high-level selection are combined, and so the less we use one, the more we use the other (and I have just criticized both).*

*This problem can be sharpened by discussions of introns and of sex, because both are phenomena that, like TEs, and like an evolutionarily productive mutation rate, provide evolvability and thus need an explanation *[16]*. In his chapter of 1990 *[16]*, Professor Doolittle provokingly writes that the existence of introns and the entire apparatus that allows for exon shuffling is in a sense more interesting than the entire part of the evolutionary process that traditional theory attempts to address. Rather than small quantitative changes, this apparatus allows for “quantum leaps” through the creation of new genes and enzymes. He then attempts to address this issue with high-level selection, but again admits that all that this selection can be expected to do is favor species that for one reason or another have lost fewer of their introns or have their introns positioned better in terms of their long-term usefulness through exon shuffling. However, the origin of the usefulness is not thus explained, and seems to be left to fortuitousness; and once we admit that the more important part of evolution is enabled fortuitously by the existence of a complex biological system, it is not clear how much of evolution is really explained or is explainable by the traditional theory anyhow.*

*Consideration of sex as a phenomenon that provides evolvability and that needs an evolutionary explanation also helps to sharpen the problem above. In the preface to the 1996 edition of his book—the book where he had argued that there are no high-level adaptations—Williams conceded that perhaps his greatest mistake regarded his discussion of sex *[48]*. Previously he had interpreted sex as a complex adaptation elaborated by individual selection. Now he admitted that he had underestimated the individual-level costs of sex; that it had long-term benefits; and that high-level selection most likely plays a role in explaining it. He now seems to treat it as an exception, aligning himself with common wisdom. But there is a point that I believe he missed: If the rule is that high-level adaptations do not exist because high-level selection is much weaker than individual-level selection, then if a certain evolved adaptation stands as an exception, appearing to be a high-level one, would we not expect it to be simple rather than complex, and of little rather than substantial individual-level costs, so that it would not strain the difference in effectiveness between the different levels of selection? Is it not a bit strange that the one case that evolutionary biologists tend to make an exception for is more weighty than all of the other traits that have been discussed in the context of the levels-of-selection debate, one that is so highly complex and advanced in its biological mechanisms of implementation, and that affects the structure and function of the organism across the scales of organization so thoroughly—indeed that defines the process of selection and inheritance (see the section “*Fundamental problems in traditional evolutionary theory: sex and interactions*”)?*

Given the paragraph above, and given the relatedness of the phenomena above in terms of them being different manifestations of the problem of evolvability, I actually agree with an earlier quote from Professor Doolittle’s work—from his famous 1980 paper with Sapienza. Discussing the possibility of explaining TEs by high-level selection, they write:

“The selective advantage represented by evolutionary adaptability seems far too remote to ensure the maintenance, let alone to direct the formation, of the DNA sequences and/or enzymatic machinery involved. A formally identical theoretical difficulty plagues our understanding of the origin of sexual reproduction, even though this process may now clearly be evolutionarily advantageous.”

I argue that the evolvability that ties together the question of sex and TEs, and that Doolittle and Sapienza concluded could not have arisen by high-level selection, is no more satisfactorily “explained” by fortuitousness.

In this paper, I have provided an alternative: the mutational writing phenotype implies evolvability directly, and this ties to a new understanding of sex and of the complex factors affecting mutation. Both sex and these complex influences on mutation become central to the process: while the shuffling of the genes creates new genetic combinations, the writing of mutations combines information from different loci and thus allows selection on individuals as complex wholes to have a hereditary effect in accord with fitness—which is actually not allowed by traditional theory in sexual populations.

*As a further means of clarification in light of this reviewer’s questions, notice further differences between my theory and previous work. Modifier theory, in addition to not relying on epistasis in the control of mutation, is split into theory of selected modifiers, which do not concern the mutation and recombination rates, and theory of “neutral” modifiers, which do (but do not themselves affect survival and reproduction). In contrast, my theory holds that the control of mutation is epistatic, and that genes participate pleiotropically both in the performing and in the writing phenotype. For example, my theory predicts that the fusion between TRIM5 and CypA *[105,107-112]* involved genes that, on the one hand, participated in the performing phenotype, and on the other hand participated in genetic activity in the germline that ultimately led to their fusion *[116]*. This kind of epistatic activity affecting mutation and performance pleiotropically has not been modeled, and it demonstrates that the control of mutation through genetic interactions is at the heart of the evolutionary process. Indeed, I would argue that the “quantum leaps” of the generation of new genes that Professor Doolittle refers to in his work *[16]* are not random, and that a gradual process of evolution involving both the writing and performing phenotypes predisposes the genetic system to produce them. Thus, this paper offers a new look on germline genetics, suggesting avenues for research not conceived of from traditional theory, with potentially intriguing implications (see, e.g., the subsections “Many writing mechanisms may exist in the sperm cells” and “De novo gene evolution may be subject to indirect natural selection through the writing phenotype”).*

As I have argued above, there is no longer a question of how the writing phenotype itself evolved, as though this question can be separated from others. Evolution occurs by the joint evolution of the writing and performing phenotypes. Biological action has always been central to mutation, and one should not look for an origin in an ns/rm core. Instead, the question becomes: How does the joint evolution of the writing and performing phenotypes exactly happen? In this paper I have merely begun to describe this process (see points on sex, on convergence, and on selection being not a passive judge of phenotypic meaning, but an active participant in its formation). To make an analogy, the “learning apparatus” of evolution is not accidental mutation running through a sieve; it is a learning apparatus that absorbs information from selection based on its abilities evolved so far and that grows along with the information that it absorbs over evolutionary time.

Despite differences between my viewpoint and Professor Doolittle’s on the topic of high-level selection, I would like to mention that I have found his publications on evolvability in the contexts abovementioned inspiring. While others have commonly either ignored the question altogether or have not even recognized that it exists, this luminary has been unique in discussing it prominently and openly and in articulating the interest for evolution that lies in it. This has been both a great inspiration for me and has also had a profound effect on my own thinking and the development of my ideas on these topics.

I would also like to mention that, in addition to his points of criticism which I have addressed above, Professor Doolittle writes that my hypothesis on sex would make it fundamental for evolution; that I have summarized a “vast number of phenomena which neoDarwinists have to stretch themselves to rationalize”; and that I do seem to be offering hope for a new conceptual framework within which to address these phenomena.

*I would like to thank him greatly for his time and effort in reading and commenting on the first and less clear draft of this manuscript. In an effort to clarify the paper, I have revised key parts of the text substantially and have added a point-by-point outline of the material discussed (see Summary section).*

## Endnotes

^**a**^ By defining the mutation that drives evolution as described in this paper, I do not mean that this mutation is nonrandom in a global sense. Events affecting mating as well as other events affecting the outcome of recombination and the writing of mutation are not predictable as a whole and provide sources of “randomness” in the sense in which this word is normally perceived. (In other words, mutation depends on the individual genotype, and the composition of the latter is, to an important degree, random.) Thus, in a global sense, the mutation that drives evolution as described in this paper is still random. However, the nature of this mutation as described here is unambiguously different from the one held by the traditional theory of evolution and nonrandom in that this mutation is caused by an organic process that is part of the evolving organism. In fact, it is the outcome of evolved and continually evolving biological system. This new concept of the mutation that drives evolution (not inclusive of all the mutations that cause disease) is further developed in the section “A more detailed look into the new theorypredictions for molecular evolution”.

^**b**^ These are interpreted as decoys because multiple investigators have occasionally observed parasites in them, and the cost of being located by a parasite is large and obvious [99].

^**c**^ This approach is not without difficulties [270], but when done with care can be very productive [271].

^**d**^ Interestingly, and in accord with the present view, as will become clearer later, the SOS response in terms of the increase in mutation rate is in fact not equal across the genome but modulated by hotspots [255].

^**e**^ To address *de novo* genes from a traditional viewpoint, it is said that Jacob did not know that there is so much transcriptional “noise”, that *de novo* genes are “sufficiently small”, and that they only need “minimal functionality” to “get started” [192]; and that with so much transcriptional noise, and minimal requirements, occasionally some pieces of neutrally-evolving junk fortuitously acquire use in the organism. But how can we be sure that the *de novo* genes are small enough, that the amount of transcriptional noise is large enough, and that opportunities for functionality, even minimal, are common enough? Say that a person has left Jerusalem by foot, and that we believe that he performs a random walk, stepping in entirely random directions forever after; and say that we all agree that he would never reach Paris in this manner, because it is too far for a random walk. Now say we observe him in Istanbul. Would we say that it makes sense that he got there, because Istanbul is closer to Jerusalem than Paris is? Or should we perhaps reexamine our assumptions about the person? This example shows that there is a severe problem of lack of quantification of the amount of chance that we call upon, not to mention such facts as that the *Poldide novo* gene arose already with an alternative splicing pattern [11]. The reason that we say that *de novo* genes must be “small enough” is not because we know that this “explanation” works, but because we have not had any other explanation up to now. In fact, *de novo* genes are small because they are new genes, and there is a trend whereby the length of a gene increases with age. This trend would be expected from the present theory, which upholds a mechanistic, gradual origin of *de novo* genes. Last but not least, according to the present theory, the fact of transcriptional promiscuity is indeed eminently relevant to the situation, but in a mechanistic way, as will be discussed later in this paper.

^**f**^ To clarify, like Jacob, I do not think that a protein can be constructed without natural selection. But if protein parts are brought from elsewhere, in a long-term evolutionary process enacted by the writing phenotype, which itself gets continuous feedback from natural selection, then it is possible that we will see here things that are contradictory to traditional notions.

^**g**^ Note that the word “selfish” in “selfish elements” comes from an analogy to human behavior, where in fact it is a key principle of economics that actions in the actors’ self-interest contribute to the economy as a whole.

## Abbreviations

cSNP: Coincident SNP; ENCODE: The Encyclopedia of DNA Elements consortium; FoSTeS: Fork stalling and template switching; LCRs: Low copy repeats; MMBIR: Microhomology-mediated break-induced replication; NAHR: Non-allelic homologous recombination; NHEJ: Non-homologous end-joining; ns/rm: The natural selection and random mutation process; RBMs: Recombination-based mechanisms; RS: Replication slippage; SDs: Segmental duplications; SNP: Single nucleotide polymorphism; SRS: Serial replication slippage; TEs: Transposable elements.

## Competing interests

The author declares that he has no competing interests.

## Authors’ information

AL graduated from Princeton University, Ph.D. in Ecology and Evolutionary Biology, and is now an Assistant Professor in the Department of Biological Sciences at Virginia Tech. He has done work in theoretical population genetics, the evolution of sex and recombination, and work at the interface of biology and theoretical computer science. He conceived this project while being a Postdoctoral Research Fellow at the Miller Institute for Basic Research in Science and the Computer Science Division, UC Berkeley.
